# Spatial separation of ribosomes and DNA in Asgard archaeal cells

**DOI:** 10.1038/s41396-021-01098-3

**Published:** 2021-08-31

**Authors:** Burak Avcı, Jakob Brandt, Dikla Nachmias, Natalie Elia, Mads Albertsen, Thijs J. G. Ettema, Andreas Schramm, Kasper Urup Kjeldsen

**Affiliations:** 1 Department of Biology, Section for Microbiology, Aarhus University, Aarhus, Denmark; 2 Laboratory of Microbiology, Wageningen University and Research, Wageningen, the Netherlands; 3 Department of Chemistry and Bioscience, Center for Microbial Communities, Aalborg University, Aalborg, Denmark; 4 Department of Life Sciences, Ben-Gurion University of the Negev, Beer Sheva, Israel; 5 Center for Electromicrobiology, Aarhus University, Aarhus, Denmark

**Keywords:** Soil microbiology, Microbial ecology, Archaeal physiology

## Abstract

The origin of the eukaryotic cell is a major open question in biology. Asgard archaea are the closest known prokaryotic relatives of eukaryotes, and their genomes encode various eukaryotic signature proteins, indicating some elements of cellular complexity prior to the emergence of the first eukaryotic cell. Yet, microscopic evidence to demonstrate the cellular structure of uncultivated Asgard archaea in the environment is thus far lacking. We used primer-free sequencing to retrieve 715 almost full-length Loki- and Heimdallarchaeota 16S rRNA sequences and designed novel oligonucleotide probes to visualize their cells in marine sediments (Aarhus Bay, Denmark) using catalyzed reporter deposition-fluorescence in situ hybridization (CARD-FISH). Super-resolution microscopy revealed 1–2 µm large, coccoid cells, sometimes occurring as aggregates. Remarkably, the DNA staining was spatially separated from ribosome-originated FISH signals by 50–280 nm. This suggests that the genomic material is condensed and spatially distinct in a particular location and could indicate compartmentalization or membrane invagination in Asgard archaeal cells.

## Introduction

The origin of the eukaryotic cell is a major unresolved puzzle in the history of life. Several lines of evidence suggest that a merger between an archaeal host [1] and an Alphaproteobacteria-related symbiont [2] constituted a key event in the evolution of the eukaryotic cell. The archaeal host was likely an ancestral Asgard archaeon, as recent phylogenomic analyses showed that the Asgard archaea superphylum (e.g., Loki-, Thor-, Odin-, Heimdall-, and Helarchaeota) comprises the closest known extant prokaryotic relatives of eukaryotes [1, 3, 4]. Genomes of Asgard archaea are also enriched in eukaryotic signature proteins (ESPs) that are homologous to eukaryotic proteins involved in membrane trafficking, vesicle formation and/or transportation, protein ubiquitinylation, and cytoskeleton formation [1, 3, 4]. The presence of these ESPs suggests that the archaeal host already possessed some building blocks of cellular complexity before the first eukaryotic cell emerged. Microscopic investigations of the first cultured Lokiarchaeon “*Candidatus* Prometheoarchaeum syntrophicum” strain MK-D1 revealed thin, sometimes branched, membrane protrusions with cytosolic connection but no visible intracellular membrane structures [5]. This provided the first glimpse into the cell biology of Lokiarchaeota. However, Asgard archaea are highly diverse [6] and the exact branching point of eukaryotes within the superphylum is still uncertain [1, 7]. Therefore, visualization of Asgard archaeal cells in the environment is essential for a comprehensive understanding of their cellular structure and morphological diversity. Before their metagenomic identification, when Lokiarchaeota were known as Marine Benthic Group B, Knittel and colleagues visualized their cells in marine sediments [8] yet methodical limitations did not allow to discern single cells and to draw any in-depth conclusion on their morphology. A recent study suggested that Loki- and Heimdallarchaeota cells from brackish lake sediment had highly diverse morphologies and cell sizes up to 12 µm, allegedly with condensed DNA [9].

Here, we visualize Loki- and Heimdallarchaeota cells from marine sediments (Aarhus Bay, Denmark) by catalyzed reporter deposition-fluorescence in situ hybridization (CARD-FISH) and super-resolution microscopy. We captured the 16S rRNA sequence diversity of these two phyla using a recently established primer-free rRNA sequencing method [10]. Asgard archaeal 16S rRNA sequence diversity is poorly covered by common 16S rRNA gene sequence primer sets [10]. The primer-free approach therefore enabled us to obtain almost full-length 16S rRNA sequences of a diverse range of Loki- and Heimdallarchaeota populating the sediments and to design two specific oligonucleotide probes with high coverage for each of the two phyla. This allowed unambiguous visualization of Loki- and Heimdallarchaeota cells using dual-probe hybridizations. Our results invariably show 1–2 µm large, coccoid cells with spatially separated DNA and riboplasm, suggesting a potential for compartmentalization or membrane invagination in Asgard archaeal cells.

## Results and discussion

**Fig. 1:**
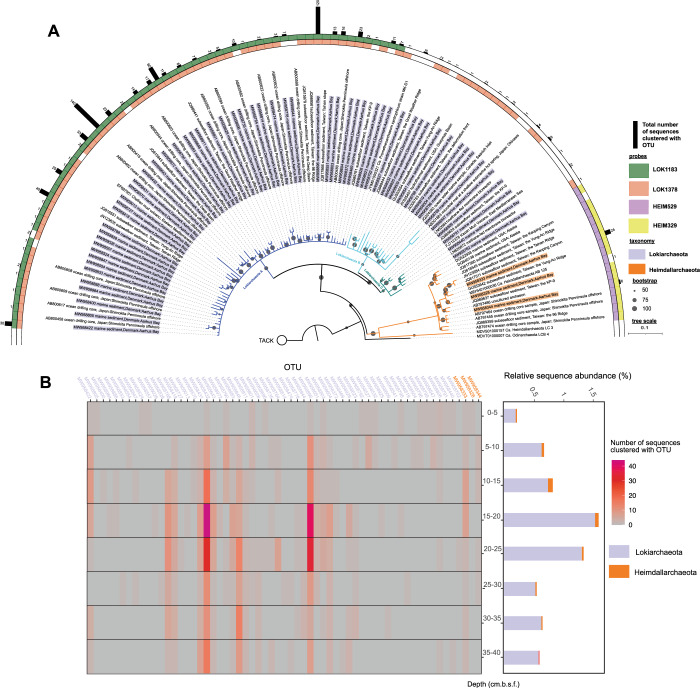
Phylogenetic analysis and depth distribution of Loki- and Heimdallarchaeota 16S rRNA sequences from Aarhus Bay sediments. **A** Maximum likelihood phylogeny of Loki- and Heimdallarchaeota operational taxonomic units (OTUs) and related sequences selected from the SILVA database (v. 132). Specificities of FISH probes and the number of sequences constituting each OTU are also depicted. TACK archaea were selected as outgroup. Bar: 0.1 substitutions per nucleotide position. **B** Heatmap and relative abundances of Loki- and Heimdallarchaeota sequences at different sediment depths.

We retrieved 684 Lokiarchaeota and 31 Heimdallarchaeota near-full-length 16S rRNA sequences from sequence libraries generated from sediment sampled at 27 m water depth in 5 cm intervals between 0 and 40 cm below seafloor (cm.b.s.f) in Aarhus Bay (Supplementary Information). The maximum relative read abundance of Lokiarchaeota was 1.6% at 15–20 cm.b.s.f. and 0.1% for Heimdallarchaeota at 10–15 cm.b.s.f. (Fig. 1). The sequences were grouped into 58 Loki- and 3 Heimdallarchaeota operational taxonomic units (OTUs) using a 98% sequence identity threshold and formed three distinct Lokiarchaeota clades and one monophyletic Heimdallarchaeota cluster (Fig. 1). The primer-free sequencing of RNA extracts enabled us to broadly sample the Asgard archaeal diversity in Aarhus Bay sediments and provided a solid database to design oligonucleotide probes for their visualization.

Based on the newly retrieved full-length sequences, we designed four novel oligonucleotide probes specifically targeting Loki- and Heimdallarchaeota 16S rRNA with high coverage (Fig. 1, Supplementary Table 1). Probe LOK1183 targets almost all sequences in Lokiarchaeota Clade A, which contains 92% of the retrieved Lokiarchaeota sequences from Aarhus Bay sediments, while probe LOK1378 targets 85% of the sequences in all three Lokiarchaeota clades. Probe HEIM329 and HEIM529 each target >97% of the retrieved Heimdallarchaeota sequences. All designed probes cover >89% sequences in their target groups in the SILVA database (v. 132). The two Lokiarchaeota probes match 5 and 10 different non-target sequences in the SILVA database (v. 132), respectively, while the Heimdallarchaeota probes have no match outside their target group. The broad coverage and high specificity suggest that our probes can also be used to detect Loki- and Heimdallarchaeota in other habitats. Furthermore, designing two probes for each phylum enabled us to identify Lokiarchaeota clade A and Heimdallarchaeota cells via double hybridizations with two distinct dyes and thus confidently distinguish true- and false-positive signals (Supplementary Fig. 1). The general archaeal probe ARC915 also targets Lokiarchaeota and thereby provided yet another control for specific hybridization of the two Lokiarchaeota-specific probes, while the non-sense probe NON338 served as the negative control. We also designed competitor probes to minimize the theoretical false-positive hybridizations with the most frequent one and two mismatches [11] in the SILVA database (v. 132) and helper probes to facilitate probe binding [12]. This comprehensive experimental design with appropriate controls enabled reliable detection of low-abundant Loki- and Heimdallarchaeota cells in Aarhus Bay sediments.

**Fig. 2:**
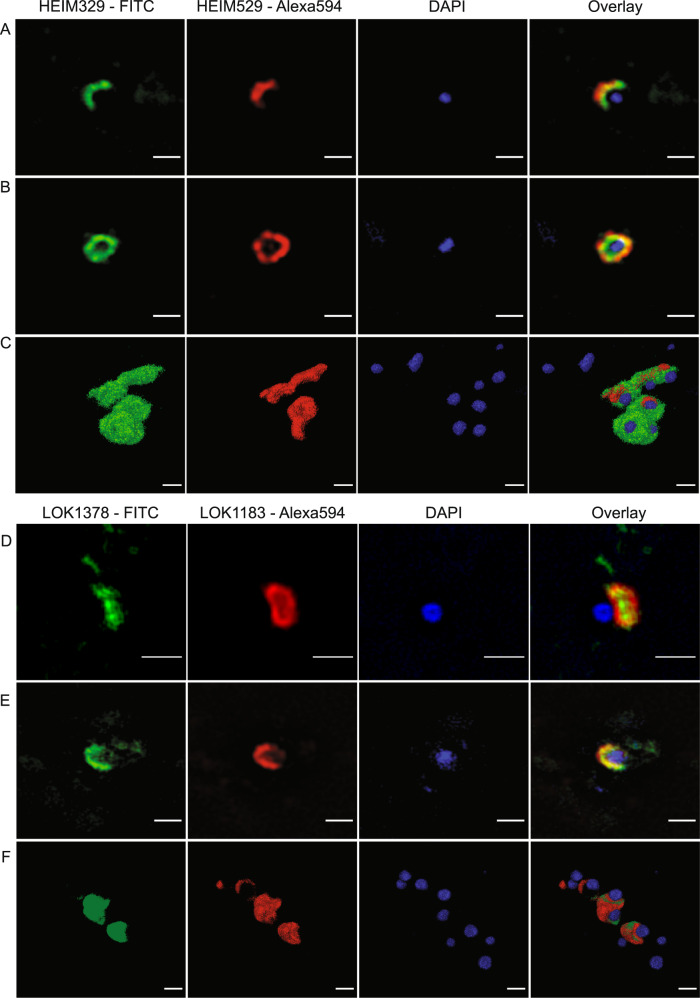
Visualization of Loki- and Heimdallarchaeota cells in Aarhus Bay sediments by CARD-FISH. Probe names and the dyes are indicated for each panel. Representative cell morphotypes were imaged in a super-resolution structured illumination microscope (SR-SIM; panels (**A**), (**B**), (**D**), (**E**)) and confocal laser scanning microscope (CLSM; panels (**C**) and (**F**)). For SR-SIM images, single slices from the center of the focal plane are shown. For CLSM images, three-dimensional (3D) surface reconstructions are depicted. All z-stack images taken in CLSM are included in Supplementary Fig. 2. 360° rotation of 3D reconstructed images are also provided in Supplementary Video. Negative and positive controls are shown in Supplementary Fig. 1 together with large ovoid and filamentous false-positive signals. Images are representative of dual labeled Lokiarchaeota (*n* = 72) and Heimdallarchaeota (*n* = 70) cells in five individual experiments using two different sediment cores taken from the same sampling site. The scale bar is 1 µm.

We used both confocal laser scanning microscopy (CLSM) and three-dimensional super-resolution structured illumination (SR-SIM) microscopy for detailed imaging of dual-labeled Loki- and Heimdallarchaeota signals. Loki- and Heimdallarchaeota cells featured coccoid shapes and often formed clusters (Fig. 2) (Supplementary Fig. 2). Based on SR-SIM imaging, Lokiarchaeota cells (*n* = 18) were 1.27 ± 0.24 µm in diameter and 1.43 ± 0.25 µm in length, while the width and the length of Heimdallarchaeota cells (*n* = 11) were 1.30 ± 0.20 µm and 1.37 ± 0.21 µm, respectively (Supplementary Table 2). In addition, we observed a few large (>3 µm) ovoid and filamentous cells, resembling some of the Lokiarchaeota morphotypes reported from lake sediment [9]; however, we never detected these cell types in double hybridizations with two probes (Supplementary Fig. 1P–R), and therefore consider them false-positives.

The DNA stain (4′,6-diamidino-2-phenylindole; DAPI) in the FISH-identified Loki- and Heimdallarchaeota cells was consistently confined to a single spherical central or lateral position (Fig. 2), corroborating the signal pattern suggested for some of the Asgard archaeal cells in lake sediments [9]. Using SR-SIM, we could image a clear gap, which separated the DNA from the ribosome-originated FISH signals with an average width of 0.18 ± 0.07 µm in Heimdallarchaeota and 0.16 ± 0.13 µm in Lokiarchaeota cells (Supplementary Table 2). The spatial separation of DNA and ribosomes in Loki- and Heimdallarchaeota cells represents an unusual observation since DAPI and FISH signals generally overlap partially or completely in prokaryotic cells [13]. Accordingly, SR-SIM imaging of benthic bacteria in Aarhus Bay sediments demonstrated the prevalence of this overlapping signal pattern (Supplementary Fig. 3). Also, the separated DNA signal observed in Loki- and Heimdallarchaeota cells appeared different from the condensed DNA formation previously described, for example, in *Escherichia coli* cells [14] and the Thaumarcheota *Cenarcheum symbiosum* [15] and *Nitrosopumilus maritimus* [16]. To corroborate this, we performed SR-SIM imaging of CARD-FISH-labeled *E.coli* and *N. maritimus* cells. Although their DNA was condensed in particular cellular locations, their FISH and DAPI signals always overlapped, indicating that their DNA and ribosomes are partially co-localized and not fully separated (Supplementary Fig. 4).

To assess whether the gap between DAPI and FISH signals was indicative of an internal membrane, we tried various dyes to stain membranes of the CARD-FISH-labeled Asgard archaeal cells (Supplementary Information). However, none of these stainings was successful, not even for the outer cell membrane, most likely because cell membranes were disintegrated during the CARD-FISH protocol. We then used wheat germ agglutinin (WGA), a lectin primarily binding to N-acetyl-D-glucosamine but also other glycoconjugates and oligosaccharides [17] to at least be able to visualize the surfaces of Loki- and Heimdallarchaeota cells. WGA consistently decorated a cell surface that enclosed the proximal FISH and DAPI signals, suggesting that both signals originated from the same single cell (Supplementary Fig. 5). The WGA staining also demonstrated extracellular structures connected to some Heimdallarchaeota cells (Supplementary Fig. 5). These structures appear different than the membrane protrusions in the first cultured Lokiarchaeon “*Ca*. P. syntrophicum”, which has a considerably smaller cell size (550 nm in diameter) and does not possess the separated DNA and ribosome signals [5]. Our observations therefore indicate diverse cellular organizations and morphotypes within Asgard archaea superphylum.

Our combined results suggest that genomic material is condensed and spatially distinct from the riboplasm within the detected Loki- and Heimdallarchaeota cells. Considering the anticipated role of Asgard archaea in eukaryogenesis, in particular the presence of ESPs potentially involved in dynamic cytoskeleton formation [18] and membrane remodeling [4, 19], the separation of DNA- and ribosome-derived signals might be indicative of cellular compartmentalization. Alternatively, the observed pattern could be the result of a membrane invagination to form a nucleoid region, similar to membrane organizations for example in Planctomycetes cells [20] or *Atribacter laminatus* [21].

Our study demonstrates the first visualization of diverse Loki- and Heimdallarchaeota cells in the marine environment and provides a protocol for reliable in situ imaging of rare microorganisms in environmental samples. Future research should address the ultrastructure of Asgard archaeal cells using electron microscopy. This would help to elucidate the cell biology of Asgard archaea and provide insights into the emergence of subcellular complexity of the eukaryotic cell.

## Supplementary information

Supplementary Information

Supplementary Fig. 1

Supplementary Fig. 2

Supplementary Fig. 3

Supplementary Fig. 4

Supplementary Fig. 5

Supplementary Table 1

Supplementary Table 2

Supplementary Video
