# Supplementary material for: Spatial separation of ribosomes and DNA in Asgard archaeal cells
Source: ISME J. 2021 Aug 31;16(2):606–10. doi: 10.1038/s41396-021-01098-3 (PMC8776820; doi:10.1038/s41396-021-01098-3)
Supplement: Supplementary file 1 — Supplementary Information [file 41396_2021_1098_MOESM1_ESM.docx]

***Supplementary Information***

**Materials and Methods**

*Sampling, RNA extraction and 16S rRNA sequencing*

Sediment cores were taken by a Rumohr Lot Corer (1) during cruises with the RV Aurora at Aarhus Bay (Denmark) station M5 (56.103333, 10.457833; water depth 27 m) in February 2018, June 2019, and March 2020. Intact cores with an overlying water phase were brought back to the lab and subsampled within 5 h upon retrieval. Subsamples for RNA extraction were collected with pre-cut 5 mL sterile plastic syringes from the sediment surface to 40 cm depth in 5 cm increments from a core taken during the February 2018 cruise. The syringe samples were snap-frozen in liquid nitrogen, placed in sterile WhirlPak bags (Madison, WI, USA), and stored at -80 ^o^C until used for RNA extraction. Subsamples for catalyzed reporter-deposition-fluorescence *in situ* hybridization (CARD-FISH) were collected with pre-cut 2.5 mL sterile plastic syringes from 0-5 cm depth of the sediment cores. Sediment samples were fixed with paraformaldehyde, which effectively preserves cell morphology (2). 4% formaldehyde was freshly prepared from paraformaldehyde powder (Sigma-Aldrich, St. Louis, MO, USA). The solution’s pH was adjusted to 7.0 and filter sterilized (0.2 μm pore-size, Sartorius, Göttingen, Germany). 2 ml sub-samples were then fixed in 1% formaldehyde at room temperature for 1 h. The fixed samples were centrifugated at 16.000 g for 5 minutes and the pellet was washed in 1X phosphate-buffer saline (PBS) three times. Afterwards, the samples were stored in a 1:1 PBS / ethanol mixture at -20 ^o^C until further processing.

For CARD-FISH control experiments, a single colony of *Escherichia coli* strain DH5 alpha was transferred into 10 ml of Luria Bertani medium and grown at 37 °C, 200 rpm until an OD_600nm_ of 0.5 was reached. *E.coli* cells were fixed and filtered on polycarbonate membrane filter as described above. Furthermore, *Nitrosopumilus maritimus* strain 1 was cultured in HEPES buffer medium (3) with 1 mM ammonia as sole energy and bicarbonate as sole carbon source. Cells in early stationary phase were fixed in 4% formaldehyde for 1h at room temperature and filtered on a 0.1 µm polycarbonate filter (Millipore, Cork, Ireland).

RNA was extracted from partially thawed sediment aliquots (3 g) using the RNeasy Power Soil total RNA kit (Qiagen, Hilden, Germany) according to the manufacturer’s protocol. Three replicate extractions were made from each sediment depth. The resultant RNA extracts (100 µL each) were treated with DNase (Ambion TURBO DNase, ThermoFisher Scientific, Waltham, MA, USA) to remove residual contaminating DNA. The absence of DNA following DNase treatment was confirmed by SYBR Green-based qPCR quantification of bacterial 16S rRNA genes. The qPCR primers, reagents, and assay conditions were described previously (4). The qPCR assay had a lower detection limit of 10 to 20 16S rRNA gene copies per µL template as evaluated from the linearity of standard curves and the level of background contamination in negative control samples.

The sequencing library was prepared according to (5, 6). Sequencing was carried out on the Illumina HiSeq platform (San Diego, CA, USA) as described in (5) with 10% denatured and diluted 20 pM PhiX Control v3 library (Illumina, San Diego, CA, USA). A detailed description of the data processing has been published previously (5). Briefly, molecular tagging of the 16S rRNA during library preparation allowed short-read sequences to be separated into read bins representing individual 16S rRNA molecules. Subsequently, *de novo* assembly of each read bin resulted in full-length sequences, which were subjected to trimming and filtering steps. All data processing scripts are available on github (<https://github.com/MadsAlbertsen/fSSU>). Finally, the sequencing efforts resulted in 684 Lokiarchaeota and 31 Heimdallarchaeota 16S rRNA gene sequences, which were deposited in GenBank with accession numbers from MW958324 to MW959038.

*Phylogenetic analysis and probe design*

Loki- and Heimdallarchaeota sequences were added to the alignment of the Silva SSURef NR 99  v.132 database (5) using the SILVA Incremental Aligner (7) and manually curated in ARB v.6.1 according to rRNA secondary structure (8). Operational taxonomic unit (OTU) clustering was performed at 98% sequence identity threshold using the SILVAngs pipeline (9). A phylogenetic tree including all Loki- and Heimdallarchaeota sequences in the database was reconstructed with the RAxML v.7 (10) maximum likelihood method (GTR-GAMMA rate distribution model, rapid bootstrap algorithm, 100 repetitions) using a 50% positional conservation filter for all archaeal sequences in SILVA database (v. 132) that selected 1450 sequence alignment positions for the analysis. The phylogenetic tree was subsequently visualized in iTOOL (11) (Fig. 1).

Oligonucleotide probes were designed using the probe design tool implemented in ARB with SILVA SSURef NR 99 v.132 (Supplementary Table S1). Hybridization conditions for these probes were optimized with varying formamide concentrations between 0 and 60% (10% increments) at 46 °C using the Aarhus Bay sediment samples from 0-5 cm.b.s.f.. The highest possible formamide concentration providing dual-labeled Lokiarchaeota (LOK1378 and LOK1183) or Heimdallarchaeota (HEIM329 and HEIM529) signal was used for further hybridizations. As negative control experiments, we used non-sense probe NON338 in second hybridizations after the first hybridization with Loki- or Heimdallarchaeota specific probes to confirm that detected Loki- and Heimdallarchaeota morphotypes are not random probe-binding or auto-fluorescent particles. As the positive control for the hybridization of Lokiarchaeota probes, we also used general archaea probe ARC915 in subsequent hybridizations (Supplementary Fig. 1).

*Catalyzed reporter deposition* in situ *hybridization (CARD-FISH)*

CARD-FISH analyses were performed according to (12) with minor modifications. Briefly, cells were detached from sediment particles using a HD2070 ultrasonication probe at 30% for 10 × 30s (Bandelin, Berlin, Germany) and filtered onto a 0.2 μm pore size polycarbonate membrane filter (Millipore, Cork, Ireland). Filter sections were embedded in 0.1% low gelling temperature agarose and endogenous peroxidases were inactivated in methanol with 0.15% H_2_O_2_ (Sigma-Aldrich, St. Louis, MO, USA) for 30 minutes. Cell walls were permeabilized in lysozyme solution (10 mg ml^-1^) (Sigma-Aldrich, St. Louis, MO, USA) at 37°C for 60 minutes. For *N. maritimus* cells*,* an additional permeabilization in achromopeptidase (60 U, 30 min) was performed. Hybridization was done with horseradish peroxidase (HRP)-labeled probes (0.5 ng DNA µl^-1^) together with helper and competitor probes at 46 °C (Biomers, Ulm, Germany). After washing at 48 °C, signals were amplified using fluorescein (FITC)- or Alexa488-labeled tyramides (1 µg ml^-1^) (Thermo Fisher, Waltham, MA, USA). For dual hybridizations, HRP from the first probe was inactivated by 3% H_2_O_2_ solution in sterile MilliQ water for 10 minutes and then the second CARD amplification was performed with Alexa594-labeled tyramides (1 µg ml^-1^) (Thermo Fisher, Waltham, MA, USA). Cells were counterstained with 4′,6-diamidino-2-phenylindole (DAPI, 1 µg ml^-1^) (Thermo Fisher, Waltham, MA, USA) and mounted in Citifluor (Electron Microscopy Sciences, Hatfield, PA, USA): Vecta Shield (Vector Laboratories, Burlingame, CA, USA) (4:1 v:v) medium. In total, we analyzed 72 Lokiarchaeota and 70 Heimdallarchaeota dual probe-labeled cells and 52 bacterial cells in five individual experiments using samples from two different sediment cores taken in June 2019 and in March 2020 from the same sampling site (Fig. 2).

*Microscopy*

Imaging was performed on a Zeiss (Oberkochen, Germany) LSM700 confocal laser scanning microscope (CLSM) and an ELYRA PS.1 three-dimensional (3D) super-resolution structured illumination microscope (SR-SIM). For CLSM imaging, 405, 488, and 555 nm lasers and Plan-Apochromat ×63/1.4 oil DIC M27 objective were used. Surface reconstructions (Fig. 2) were performed in Zen Black software (Carl Zeiss Microimaging, Jena, Germany) using all z-stack images (Supplementary Fig. 2) for a particular channel. For SR-SIM, stained filters were mounted on #1.5 high-resolution coverslips (Marienfeld, Lauda-Königshofen, Germany). Thin z-sections (0.11 μm) of high-resolution images were collected in 3 rotations for each channel using a ×63/1.4 oil objective and 405, 488, 561, and 647 nm lasers. For all datasets, image reconstruction and channel alignment were performed in ZEN Black software.

*Membrane staining*

To stain the membranes of FISH-stained Loki- and Heimdallarchaeota cells, we used Nile Red (Sigma-Aldrich, St. Louis, MO, USA) and FM 4-64 dye (N-(3-Triethylammoniumpropyl)-4-(6-(4-(Diethylamino) Phenyl) Hexatrienyl) Pyridinium Dibromide) (Thermo Fisher, Waltham, MA, USA). After dual-hybridization, filter sections were incubated with Nile Red (2.0 µg ml^-1^) or FM 4-64 (3.0 µg ml^-1^) for 10 minutes at room temperature and washed in MilliQ water.

*Wheat Germ Agglutinin (WGA) staining*

After CARD-FISH, filter sections were incubated with WGA conjugated to Alexa647 (5.0 µg ml^-1^) (Thermo Fisher, Waltham, MA, USA) in Hanks’ Balanced Salt solution (Sigma-Aldrich, St. Louis, MO, USA) for 10 minutes at room temperature. Samples were mounted with DAPI–Fluoromount-G medium (SouthernBiotech, Birmingham, AL, USA) and were imaged by super-resolution structured illumination microscopy (SR-SIM). WGA signals obtained in FISH- and DAPI-positive cells were at least two folds higher than background staining, confirming labeling specificity (Supplementary Fig. 3).

*Cell measurements*

All measurements were performed in ZEN Black software using the SR-SIM images. Cell width was measured from the beginning of the FISH signal to the end of the DAPI signal, based on a line intensity profile of both channels, taken from the center of the labels across the narrow axis of the cell. Gap between DAPI and FISH staining was determined as the distance between FISH signal decay and the beginning of DAPI signal. Cell length was determined based on a line intensity profile drawn along the long axis of the FISH signal (Supplementary Table 2).

**References**

1. Meischner D, Rumohr J. A light-weight high-momentum gravity corer for subaqueous sediments. *Senckenb. Marit*. 1974;6(1):105-17.

2. Chao Y, Zhang T. Optimization of fixation methods for observation of bacterial cell morphology and surface ultrastructures by atomic force microscopy. *Appl Microbiol Biotechnol*. 2011;92(2):381-92.

3. Martens-Habbena W, Berube PM, Urakawa H, de la Torre JR, Stahl DA. Ammonia oxidation kinetics determine niche separation of nitrifying archaea and bacteria. *Nature*. 2009;461(7266):976-9.

4. Starnawski P, Bataillon T, Ettema TJG, Jochum LM, Schreiber L, Chen X, *et al.* Microbial community assembly and evolution in subseafloor sediment. *Proc. Natl. Acad. Sci. U.S.A*. 2017;114(11):2940-5.

5. Karst SM, Dueholm MS, McIlroy SJ, Kirkegaard RH, Nielsen PH, Albertsen M. Retrieval of a million high-quality, full-length microbial 16S and 18S rRNA gene sequences without primer bias. *Nat. Biotechnol*. 2018;36(2):190-5.

6. Karst SM, Dueholm MS, McIlroy SJ, Kirkegaard RH, Nielsen PH, Albertsen M. RNA based library preparation for high through-put full-length small ribosomal RNA sequencing on the Illumina MiSeq and HiSeq platforms. *protocolsio*. 2017; https://dx.doi.org/10.17504/protocols.io.h2rb8d6

7. Pruesse E, Peplies J, Glöckner FO. SINA: Accurate high-throughput multiple sequence alignment of ribosomal RNA genes. *Bioinformatics*. 2012;28(14):1823-9.

8. Ludwig W, Strunk O, Westram R, Richter L, Meier H, Yadhukumar, *et al*. ARB: a software environment for sequence data. *Nucleic Acids Res*. 2004;32(4):1363-71.

9. Quast C, Pruesse E, Yilmaz P, Gerken J, Schweer T, Yarza P, *et al*. The SILVA ribosomal RNA gene database project: improved data processing and web-based tools. *Nucleic Acids Res*. 2012;41:D590-6.

10. Stamatakis A. RAxML-VI-HPC: maximum likelihood-based phylogenetic analyses with thousands of taxa and mixed models. *Bioinformatics*. 2006;22(21):2688-90.

11. Letunic I, Bork P. Interactive Tree Of Life (iTOL) v4: recent updates and new developments. *Nucleic Acids Res*. 2019;47:W256-9.

12. Ishii K, Mußmann M, MacGregor BJ, Amann R. An improved fluorescence *in situ* hybridization protocol for the identification of bacteria and archaea in marine sediments. *FEMS Microbiol. Ecol*. 2004;50(3):203-13.

**Supplementary figures, tables, and videos**

**Supplementary Fig. 1** Positive and negative control experiments for CARD-FISH hybridization and visualization of loki- and heimdallarchaeotal cells. **A-K** Non-sense probe NON338 was used in second hybridizations after the first hybridization with Loki- or Heimdallarchaeota specific probes to confirm that detected Loki- and Heimdallarchaeota morphotypes are not random probe-binding or auto-fluorescent particles. **L-O** As positive control for the hybridizations with Lokiarchaeota specific probes, general archaea probe ARC915 was used in second hybridizations. Note that ARC915 does not target Heimdallarchaeota. **P-R** Potential false-positive signals lacking double hybridization with the second probe, which resembles large ovoid and filamentous cells detected in Salcher et al. *mSphere,* 2020**.** Three-dimensional (3D) surface reconstructions from confocal laser scanning microscope (CLSM) imaging are depicted. All z-stack images for true-positive loki- and heimdallarchaeotal cells are included in Supplementary Fig. 2. Probe names and dyes for each panel are included. The scale bar is 1 µm.

**Supplementary Fig. 2** All z-stack images that were used to make three-dimensional surface reconstruction of true-positive Loki- and Heimdallarchaeota cells that are shown in corresponding panels from Fig. 2 (F2) and Supplementary Fig. 1 (SF1). Overlay of FITC, Alexa594, and DAPI are depicted. The scale bar is 1 µm.

**Supplementary Fig. 3** Super-resolution structured illumination microscopy (SR-SIM) imaging of representative bacterial cells in Aarhus Bay sediments (C-F) and their comparison to Asgard archaeal cells (A-B). Single slices from the center of the focal plane are shown. Probe names and the dyes are indicated for each panel together with the intensity line profile of DAPI and FISH signals. The gap between FISH and DAPI signals in Asgard archaeal cells are highlighted. Dashed lines indicate the position at which the fluorescence intensity line profiles were recorded. Scale bar is 1 µm.

**Supplementary Fig. 4** Super-resolution structured illumination microscopy (SR-SIM) imaging of condensed DNA formation in *Escherichia coli* (A-B) and *Nitrosopumilus maritimus* cells (C-D). Single slices from the center of the focal plane are shown. Probe names and the dyes are indicated for each panel together with the intensity line profile of DAPI and FISH signals. Dashed lines indicate the position at which the fluorescence intensity profiles were recorded.

**Supplementary Fig. 5** Wheat germ agglutinin (WGA) staining of dual-labeled Loki- and Heimdallarchaeota cells. Probe or staining names and the dyes are indicated for each panel. Extracellular structures connected to the surfaces of Heimdallarchaeota cells are indicated with arrows. The axes that were used to measure cell sizes are demonstrated in dashed lines. Imaging was performed in super-resolution structured illumination microscopy (SR-SIM). Shown are single slice images taken from the center of the focal plane. The scale bar is 1 µm.

**Supplementary Table 1** Oligonucleotide probes used in this study. **A** Names, target groups, sequences, and formamide concentrations for newly designed Loki- and Heimdallarchaeota probes. **B** Number of the sequences in the target groups and the hits are shown together with non-target hits at 0,1 and 2 mismatches. For LOK1183 and LOK1378, only the sequences which contain the probe-target regions in SILVA database v132 (Quast et al. 2013) are considered. **C** Sequences and formamide concentrations for helper and competitor probes.

**Supplementary Table 2** Measurement of **A** Heimdallarchaeota and **B** Lokiarchaeota cell sizes. Imaging was performed by super-resolution structured illumination microscopy (SR-SIM). Cell width was measured from the beginning of the FISH signal to the end of the DAPI signal, based on a line intensity profile taken along the narrow axis of the cell. Distance between DAPI and FISH staining was determined as the distance between FISH signal decay and the beginning of DAPI signal. The length was measured along the long axis of the FISH signal. The axes that were used for cell measurements were demonstrated in Supplementary Fig. 5. For the cells with central DAPI signals, only total width and total length are shown. All values are reported in µm.

**Supplementary Video** 360° rotation of three-dimensional surface reconstruction of Loki- and Heimdallarchaeota images in Fig 2. The image series is rotated along the y axis.
