# Supplementary material for: Spatial separation of ribosomes and DNA in Asgard archaeal cells
Source: ISME J. 2021 Aug 31;16(2):606–10. doi: 10.1038/s41396-021-01098-3 (PMC8776820; doi:10.1038/s41396-021-01098-3)
Supplement: Supplementary file 2 — Supplementary Fig. 1 [file 41396_2021_1098_MOESM2_ESM.pdf]

Supplementary Fig. 1 Positive and negative control experiments for CARD-FISH hybridization and visualization of loki- and heimdallarchaeotal cells. **A-K** Non-sense probe NON338 was used in second hybridizations after the first hybridization with Loki- or Heimdallarchaeota specific probes to confirm that detected Loki- and Heimdallarchaeota morphotypes are not random probe-binding or auto-fluorescent particles. **L-O** As positive control for the hybridizations with Lokiarchaeota specific probes, general archaea probe ARC915 was used in second hybridizations. Note that ARC915 does not target Heimdallarchaeota. **P-R** Potential false-positive signals lacking double hybridization with the second probe, which resembles large ovoid and filamentous cells detected in Salcher et al. *mSphere*, 2020. Three-dimensional (3D) surface reconstructions from confocal laser scanning microscope (CLSM) imaging are depicted. All z-stack images for true-positive loki- and heimdallarchaeotal cells are included in Supplementary Fig. 2. Probe names and dyes for each panel are included. The scale bar is 1 μm.

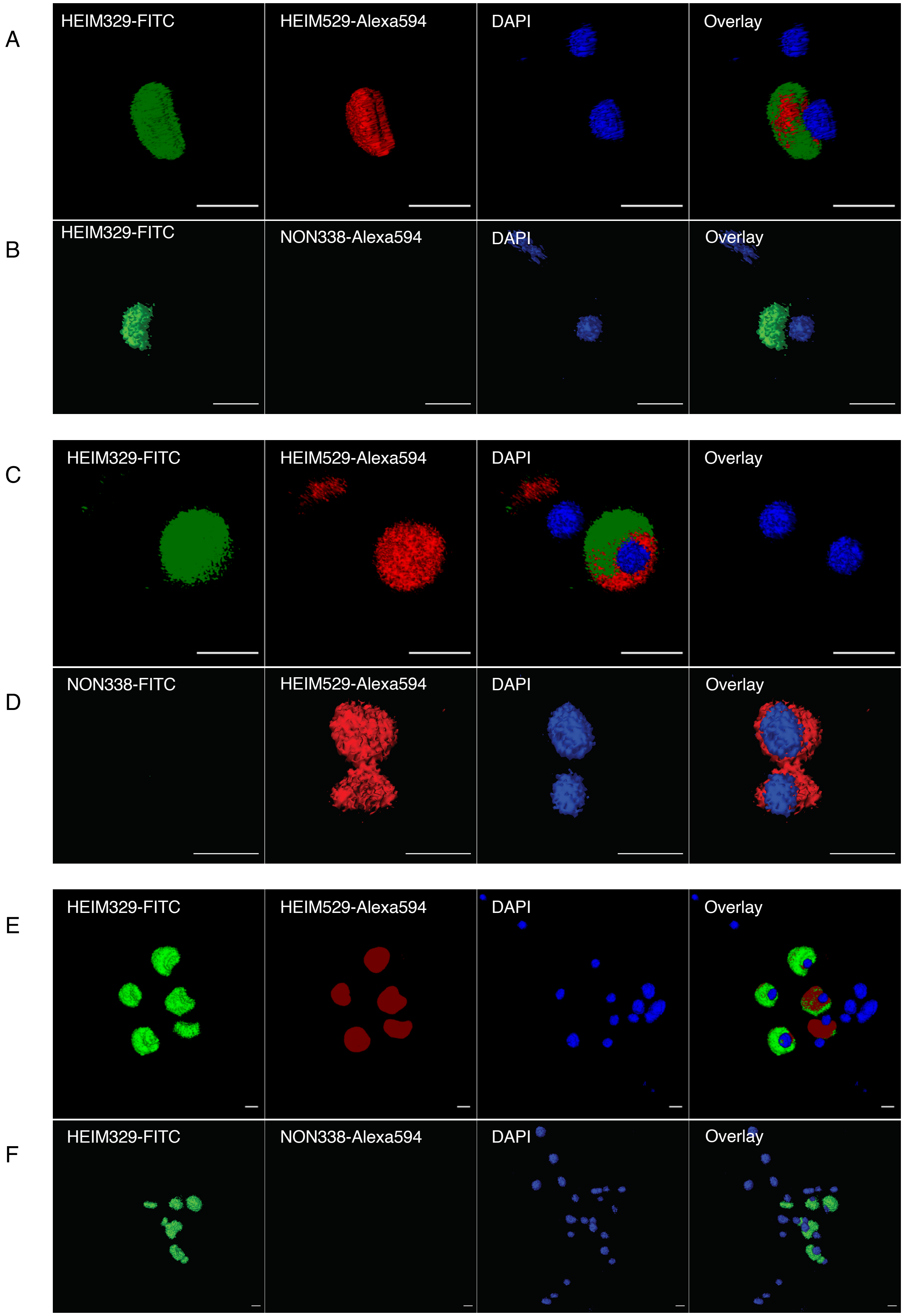
A
HEIM329-FITC
HEIM529-Alexa594
DAPI
Overlay
B
HEIM329-FITC
NON338-Alexa594
DAPI
Overlay
C
HEIM329-FITC
HEIM529-Alexa594
DAPI
Overlay
D
NON338-FITC
HEIM529-Alexa594
DAPI
Overlay
E
HEIM329-FITC
HEIM529-Alexa594
DAPI
Overlay
F
HEIM329-FITC
NON338-Alexa594
DAPI
Overlay

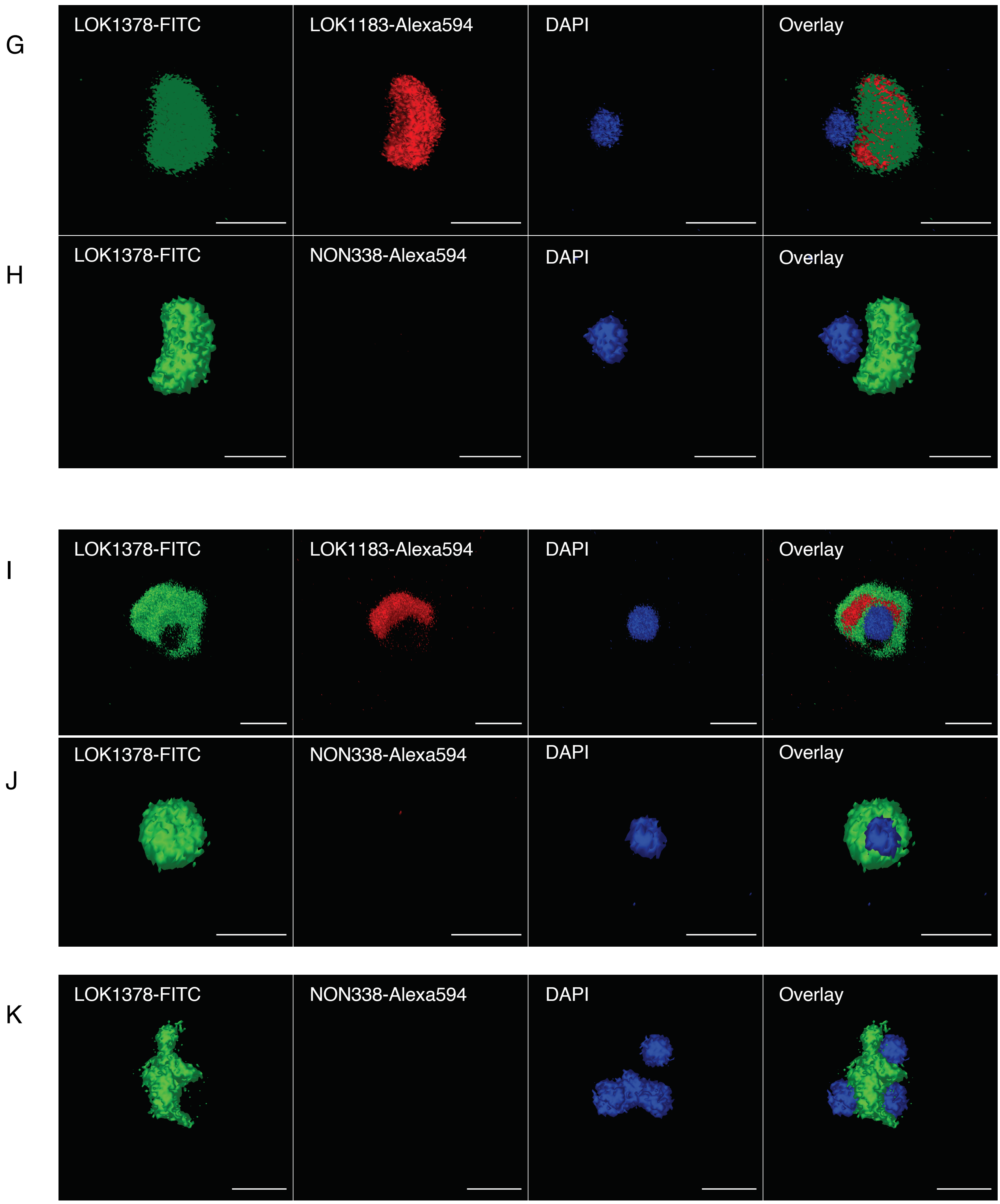
G
LOK1378-FITC
LOK1183-Alexa594
DAPI
Overlay
H
LOK1378-FITC
NON338-Alexa594
DAPI
Overlay
I
LOK1378-FITC
LOK1183-Alexa594
DAPI
Overlay
J
LOK1378-FITC
NON338-Alexa594
DAPI
Overlay
K
LOK1378-FITC
NON338-Alexa594
DAPI
Overlay

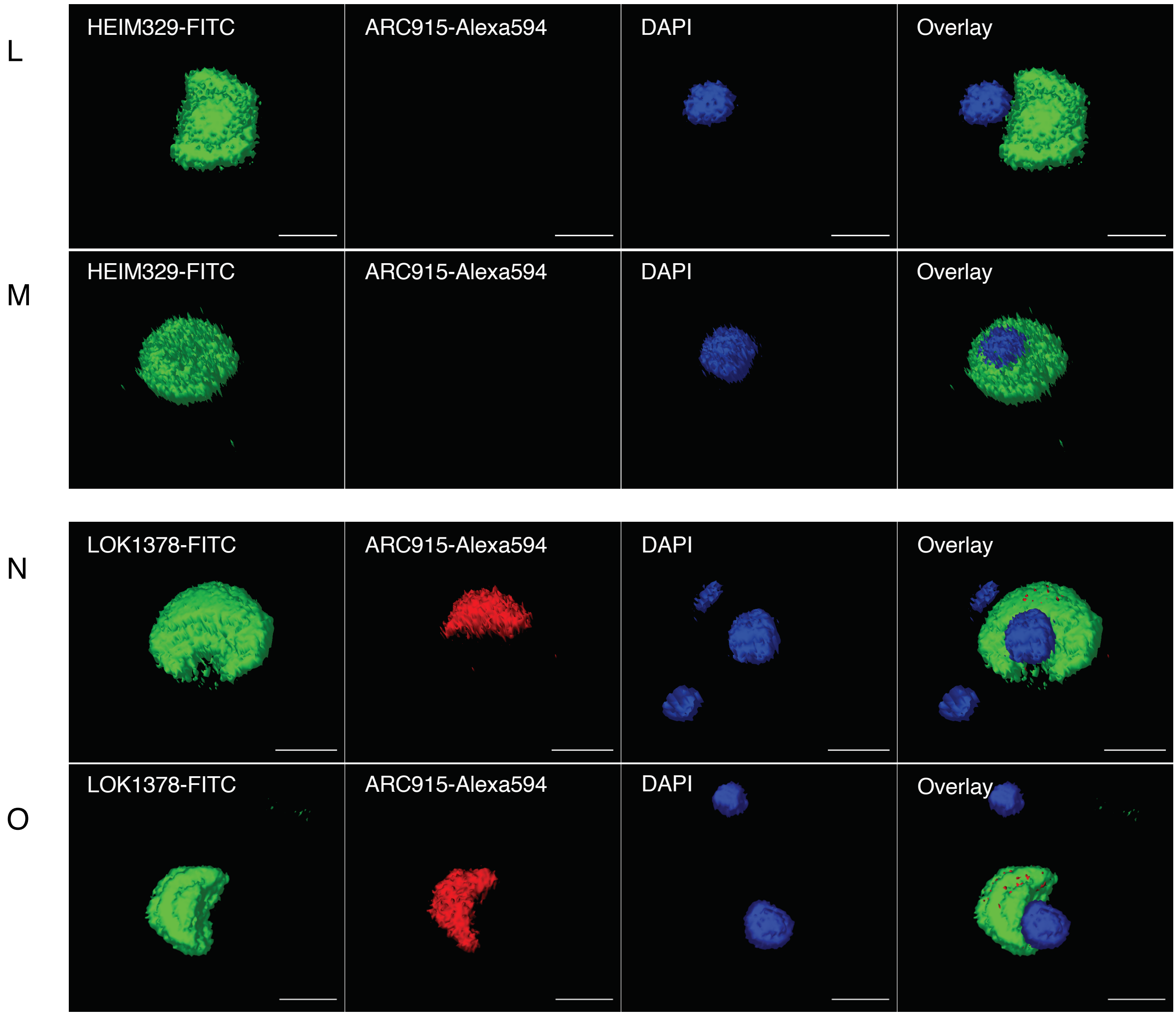
L
HEIM329-FITC
ARC915-Alexa594
DAPI
Overlay
M
HEIM329-FITC
ARC915-Alexa594
DAPI
Overlay
N
LOK1378-FITC
ARC915-Alexa594
DAPI
Overlay
O
LOK1378-FITC
ARC915-Alexa594
DAPI
Overlay

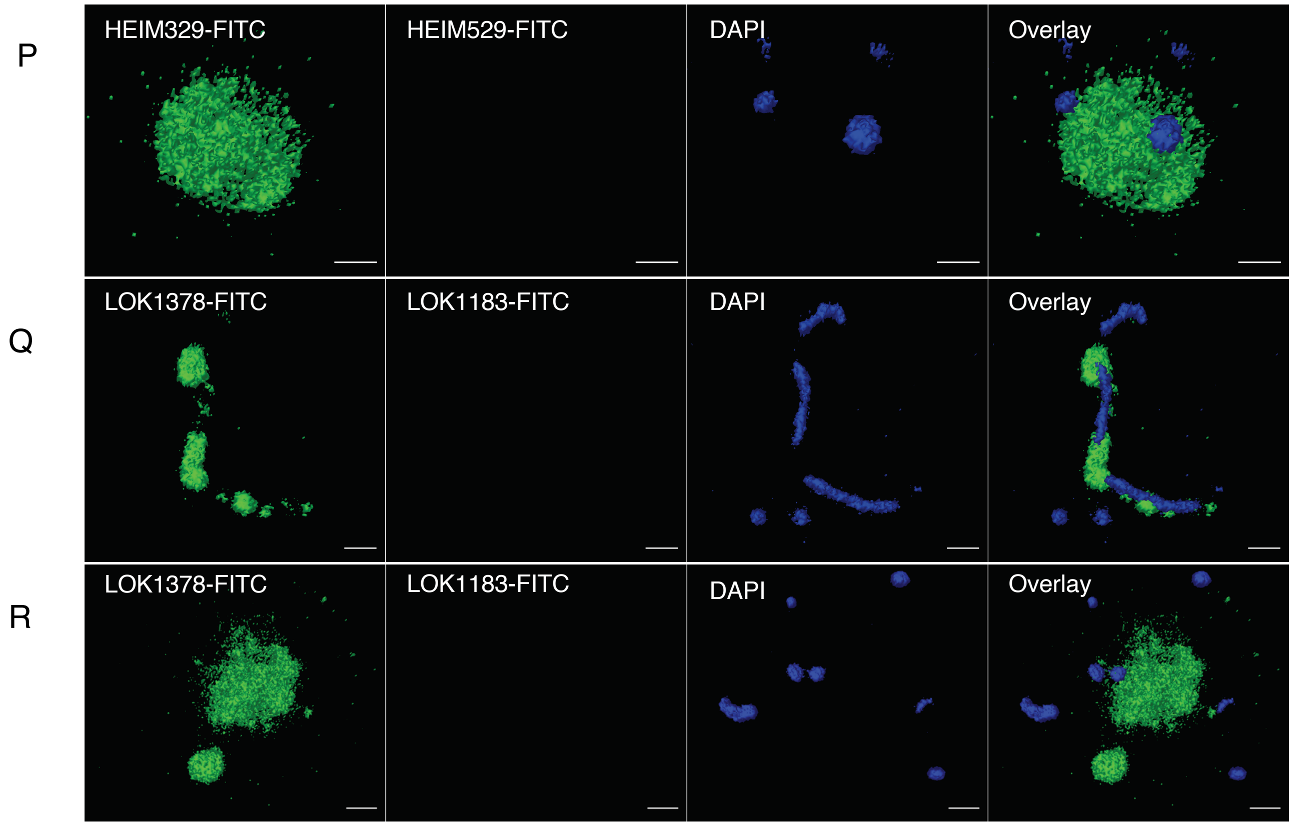
P
HEIM329-FITC
HEIM529-FITC
DAPI
Overlay
Q
LOK1378-FITC
LOK1183-FITC
DAPI
Overlay
R
LOK1378-FITC
LOK1183-FITC
DAPI
Overlay
