# Supplementary material for: Spatial separation of ribosomes and DNA in Asgard archaeal cells
Source: ISME J. 2021 Aug 31;16(2):606–10. doi: 10.1038/s41396-021-01098-3 (PMC8776820; doi:10.1038/s41396-021-01098-3)

Supplementary Fig. 2 All z-stack images that were used to make three-dimensional surface reconstruction of true-positive Loki- and Heimdallarchaeota cells that are shown in corresponding panels from Fig. 2 (F2) and Supplementary Fig. 1 (SF1). Overlay of FITC, Alexa594, and DAPI are depicted. The scale bar is 1 μm.

F2

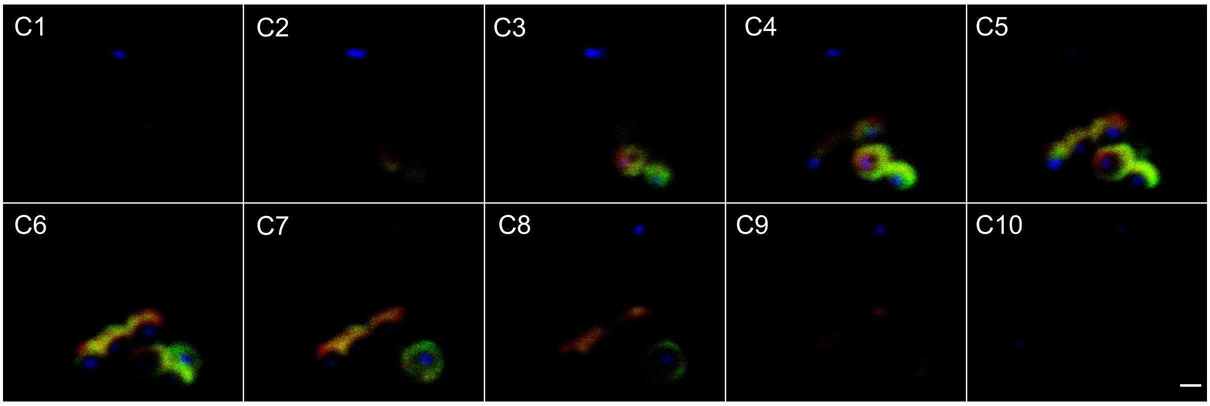
C1
C2
C3
C4
C5
C6
C7
C8
C9
C10

F2

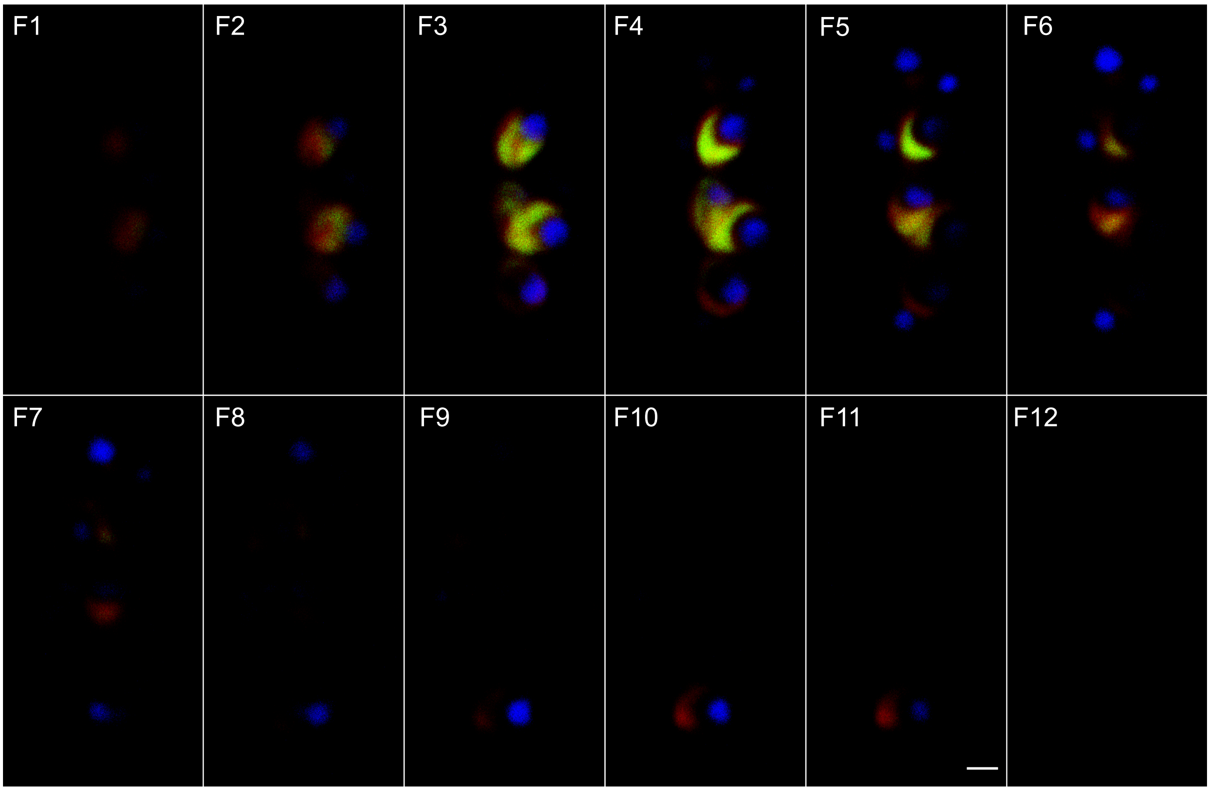
F1
F2
F3
F4
F5
F6
F7
F8
F9
F10
F11
F12

SF1

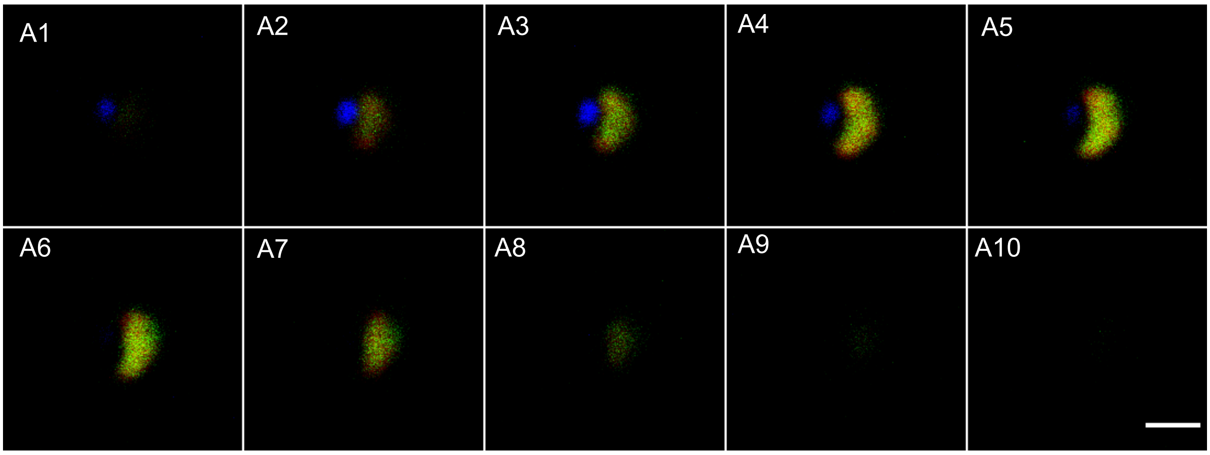
A1
A2
A3
A4
A5
A6
A7
A8
A9
A10

SF1

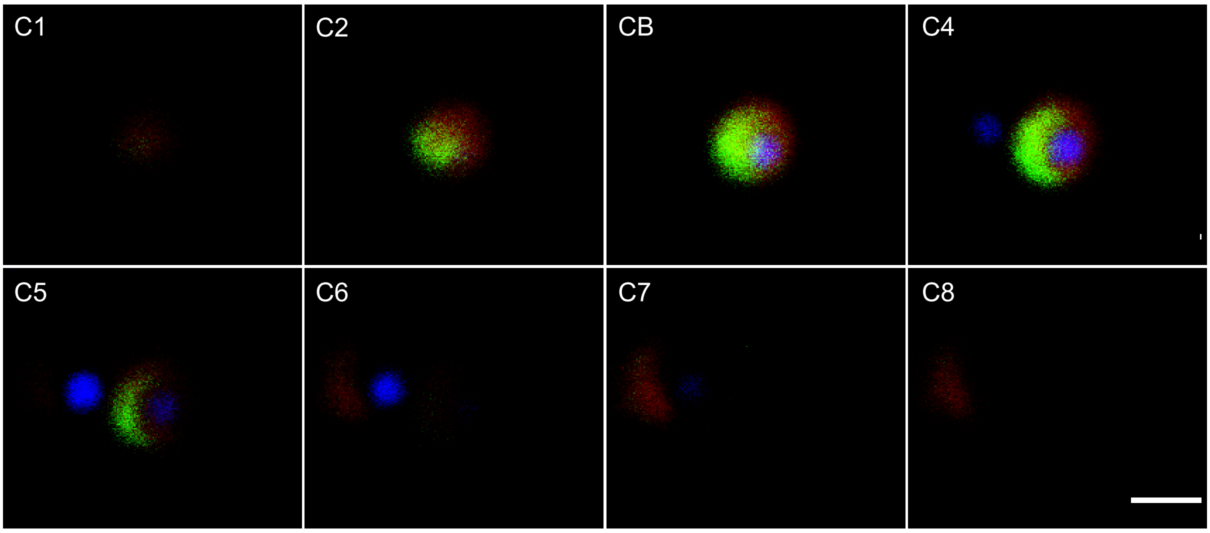

SF1

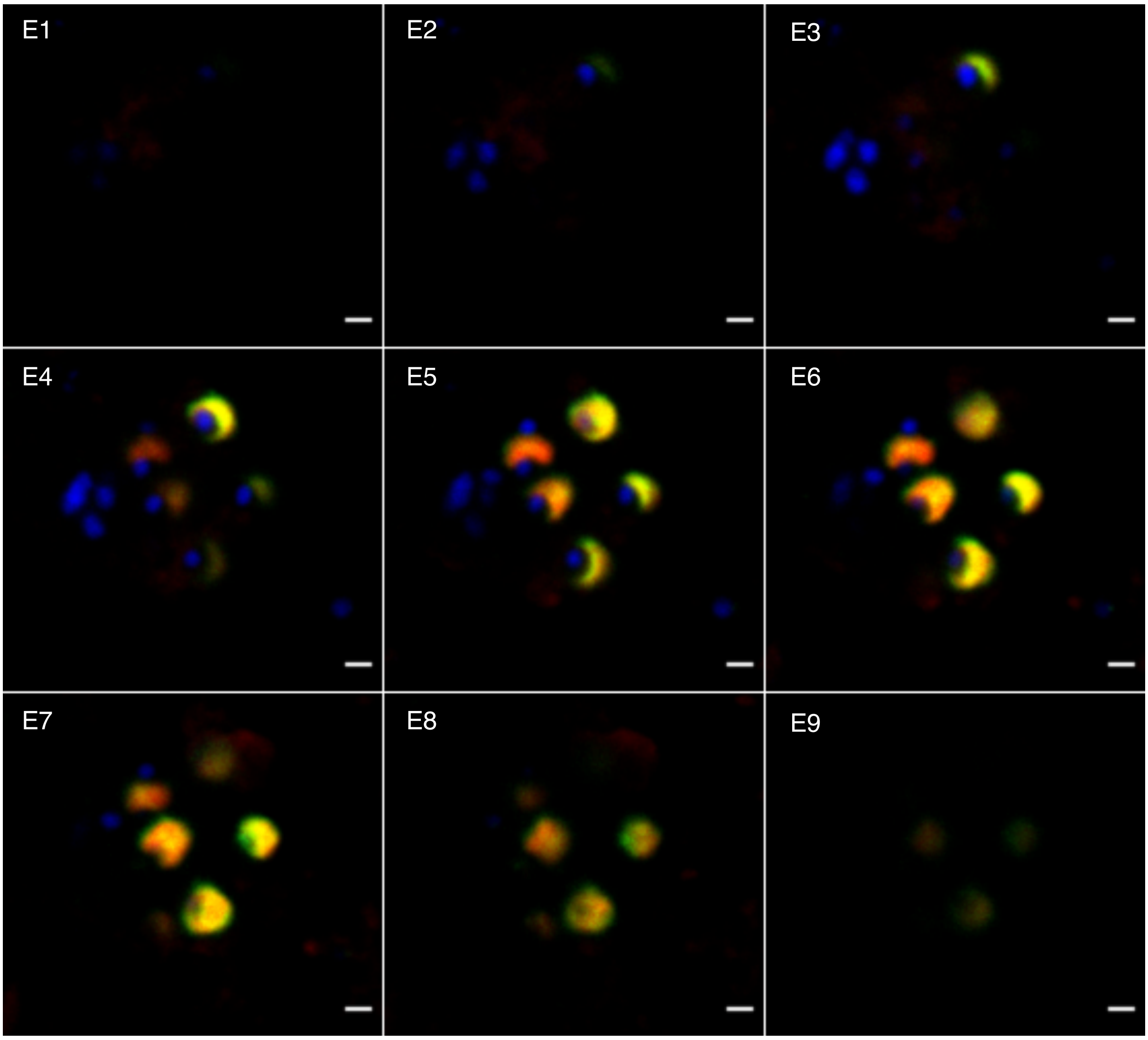

SF1

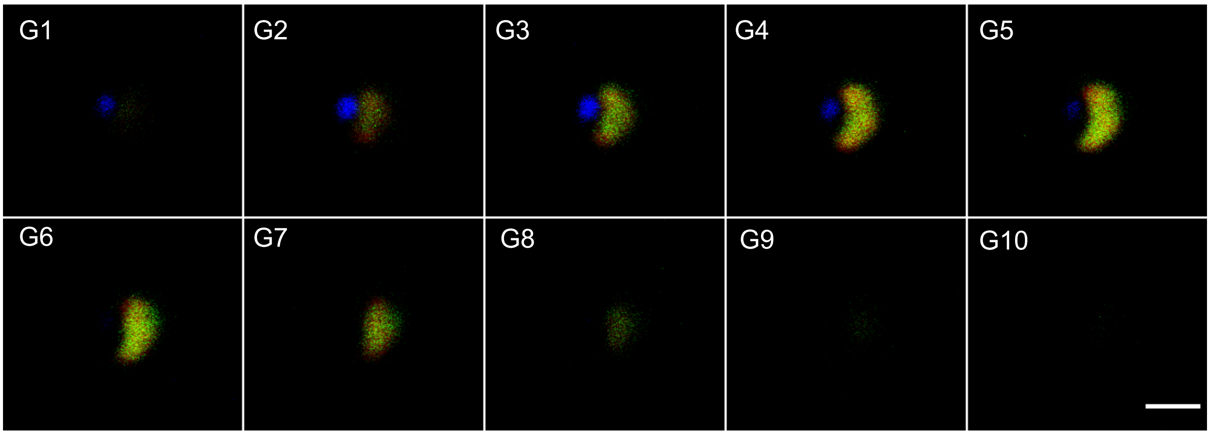

SF1

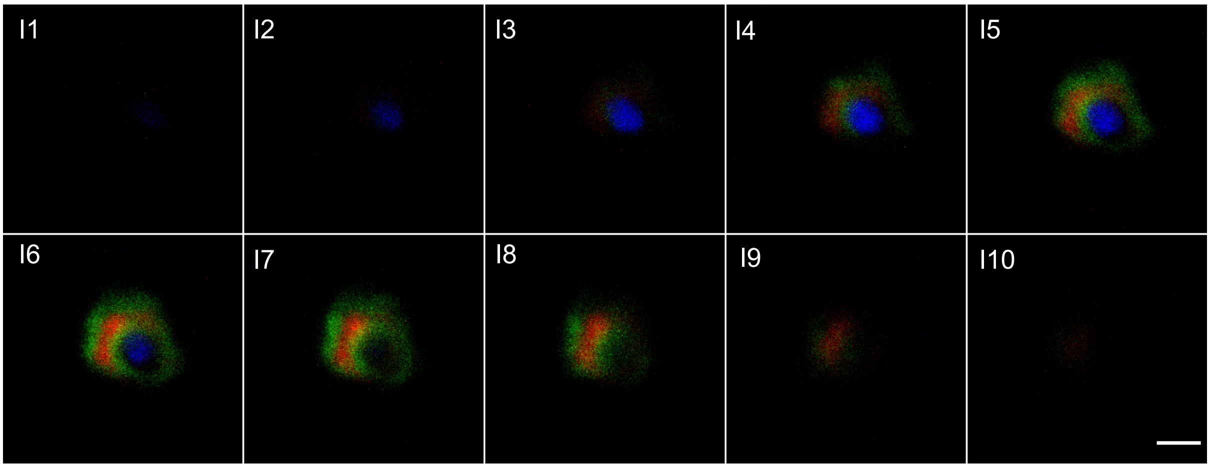

Supplement: Supplementary file 3 — Supplementary Fig. 2 [file 41396_2021_1098_MOESM3_ESM.pdf]
