# Supplementary material for: Spatial separation of ribosomes and DNA in Asgard archaeal cells
Source: ISME J. 2021 Aug 31;16(2):606–10. doi: 10.1038/s41396-021-01098-3 (PMC8776820; doi:10.1038/s41396-021-01098-3)
Supplement: Supplementary file 4 — Supplementary Fig. 3 [file 41396_2021_1098_MOESM4_ESM.pdf]

Supplementary Figure 3. Super-resolution structured illumination microscopy (SR-SIM) imaging of representative bacterial cells in Aarhus Bay sediments (C-F) and their comparison to Asgard archaeal cells (A-B). Single slices from the center of the focal plane are shown. Probe names and the dyes are indicated for each panel together with the intensity line profile of DAPI and FISH signals. The gap between FISH and DAPI signals in Asgard archaeal cells are highlighted. Dashed lines indicate the position at which the fluorescence intensity line profiles were recorded. Scale bar is 1 μm.

A

B

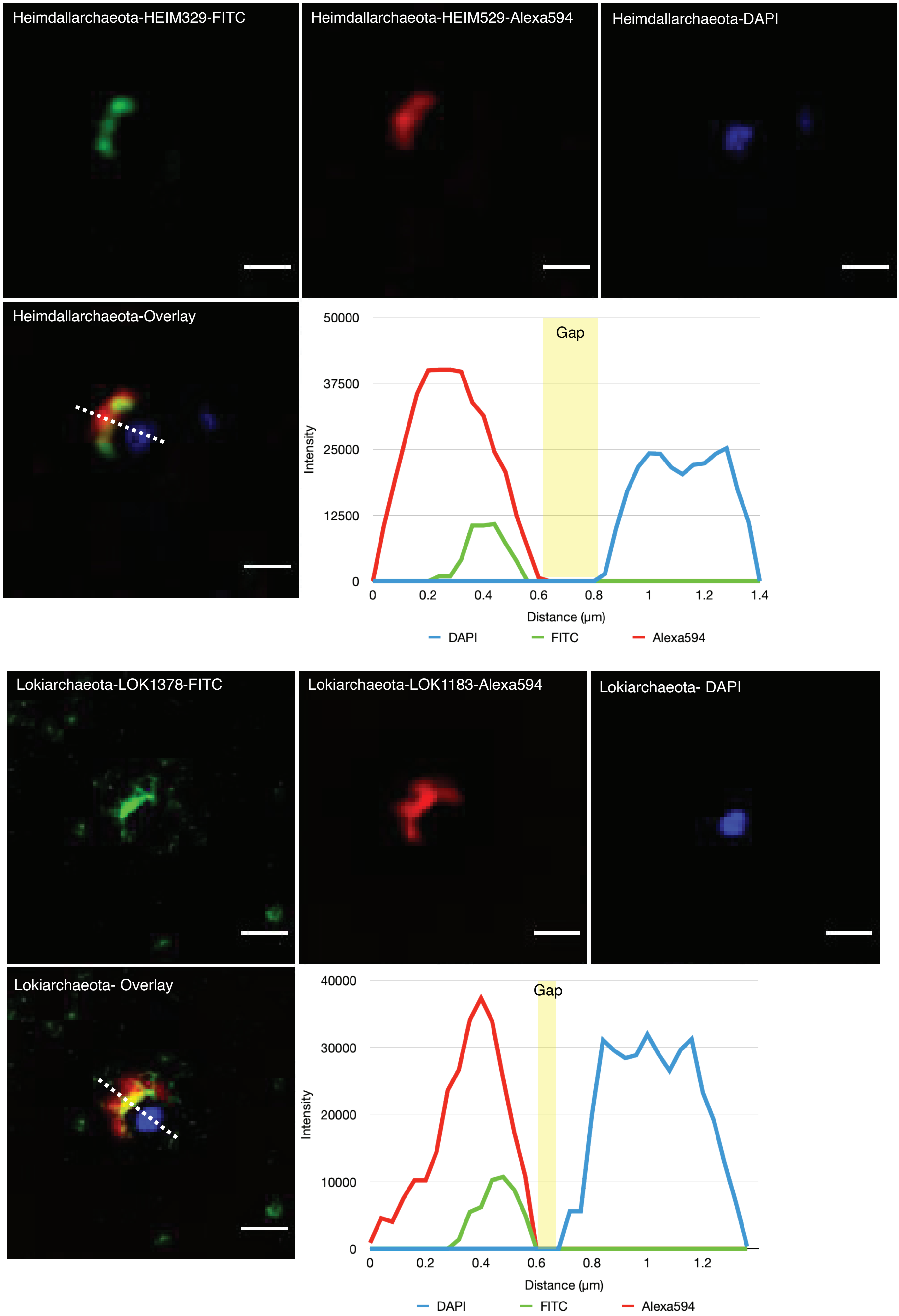

Heimdallarchaeota-HEIM329-FITC
Heimdallarchaeota-HEIM529-Alexa594
Heimdallarchaeota-DAPI
Heimdallarchaeota-Overlay
Gap
Intensity
50000
37500
25000
12500
0
0
0.2
0.4
0.6
0.8
1
1.2
1.4
Distance (μm)
DAPI
FITC
Alexa594
Lokiarchaeota-LOK1378-FITC
Lokiarchaeota-LOK1183-Alexa594
Lokiarchaeota- DAPI
Lokiarchaeota- Overlay
Gap
Intensity
40000
30000
20000
10000
0
0
0.2
0.4
0.6
0.8
1
1.2
Distance (μm)
DAPI
FITC
Alexa594

C

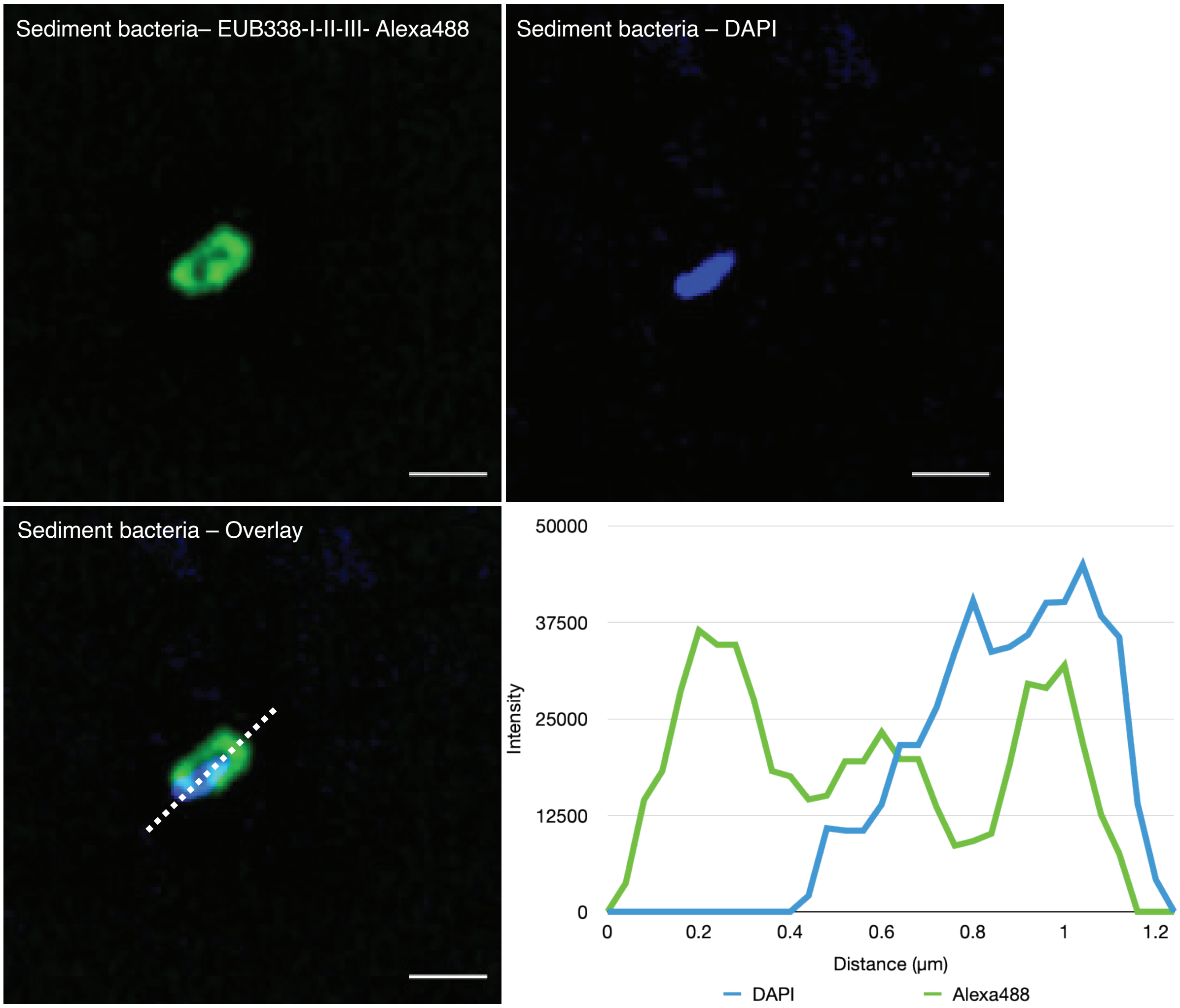
Sediment bacteria– EUB338-I-II-III- Alexa488
Sediment bacteria – DAPI
Sediment bacteria – Overlay
50000
37500
25000
12500
0
Intensity
0
0.2
0.4
0.6
0.8
1
1.2
Distance (μm)
DAPI
Alexa488

D

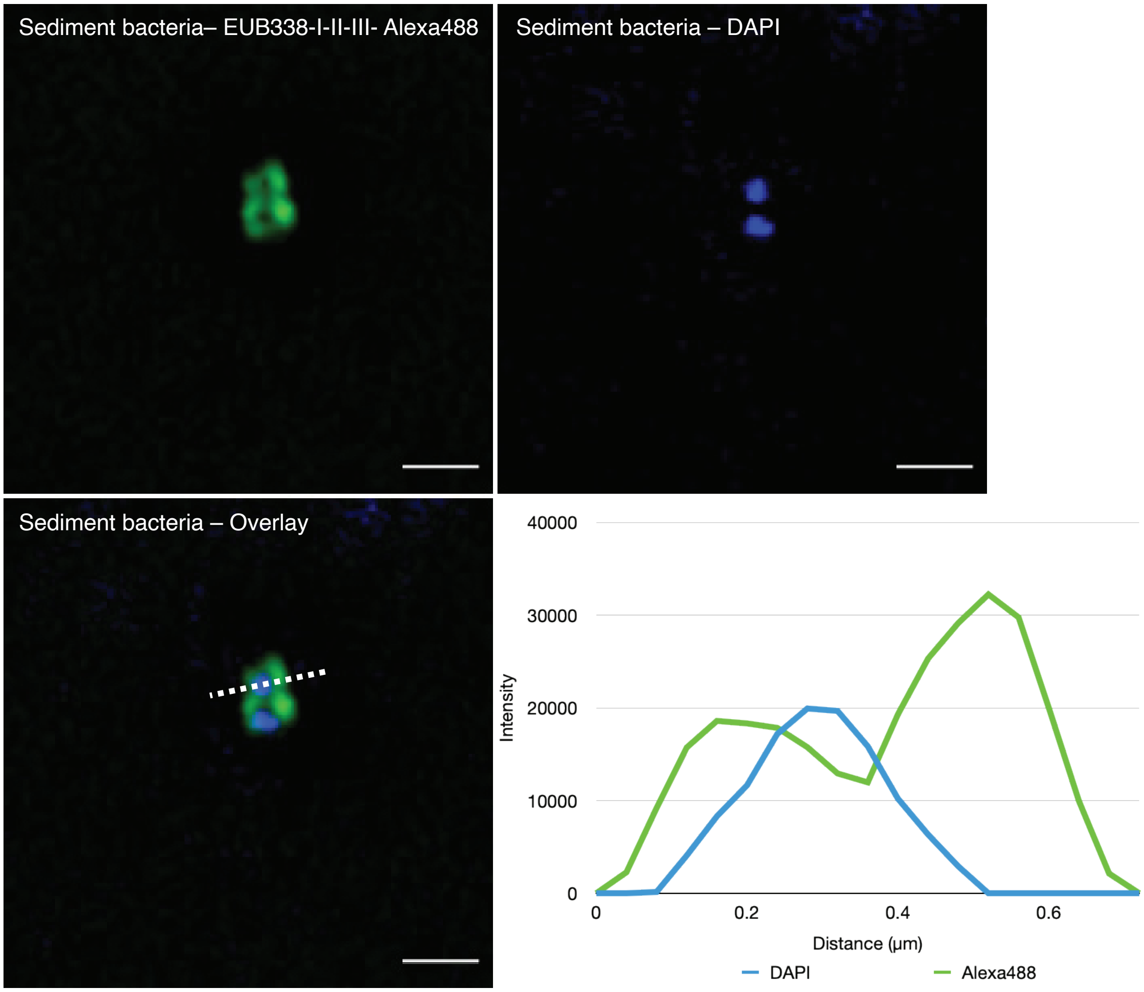
Sediment bacteria– EUB338-I-II-III- Alexa488
Sediment bacteria – DAPI
Sediment bacteria – Overlay
40000
30000
20000
10000
0
Intensity
0
0.2
0.4
0.6
Distance (μm)
DAPI
Alexa488

E

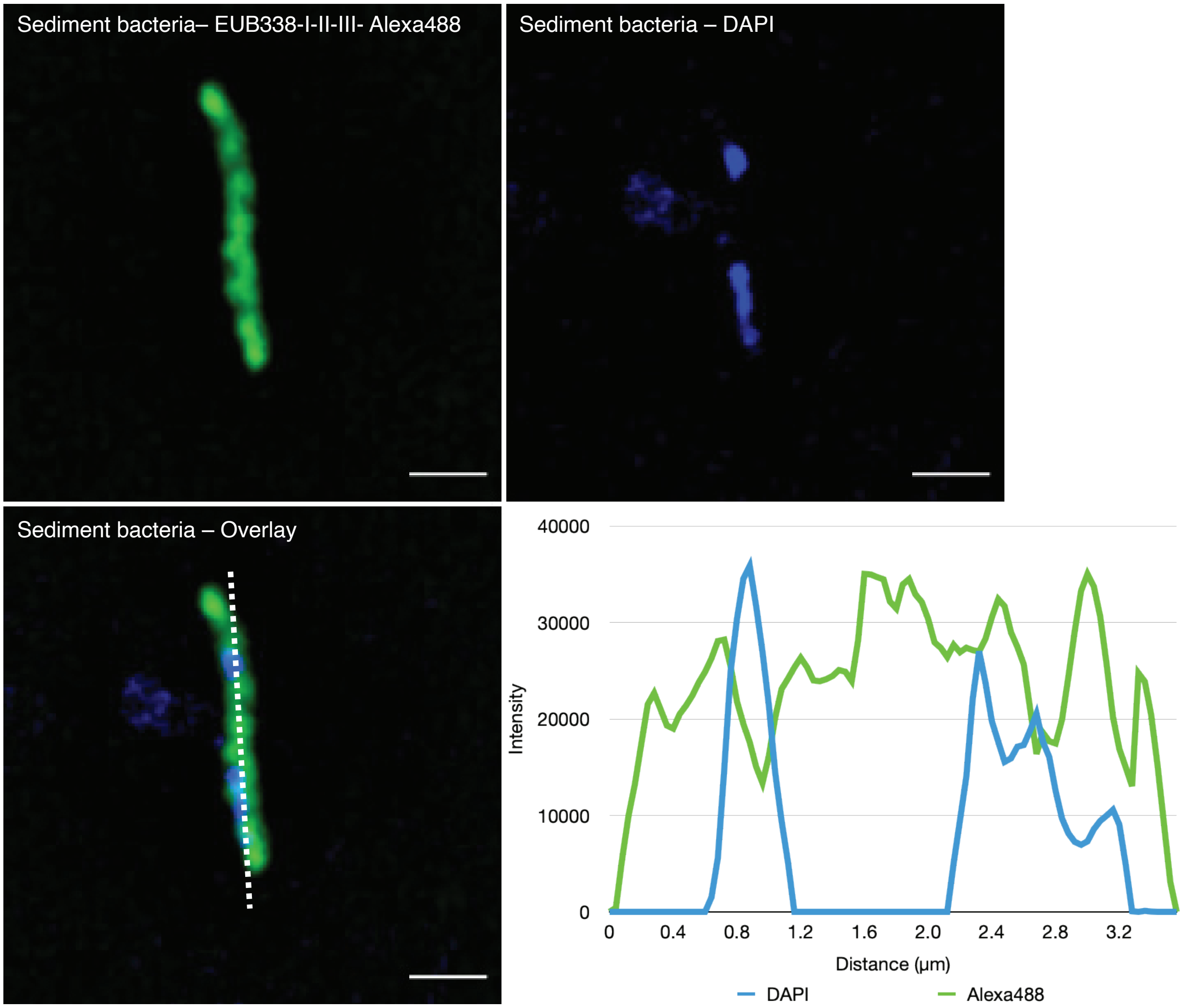
Sediment bacteria– EUB338-I-II-III- Alexa488
Sediment bacteria – DAPI
Sediment bacteria – Overlay
40000
30000
20000
10000
0
Intensity
0
0.4
0.8
1.2
1.6
2.0
2.4
2.8
3.2
Distance (μm)
DAPI
Alexa488

F

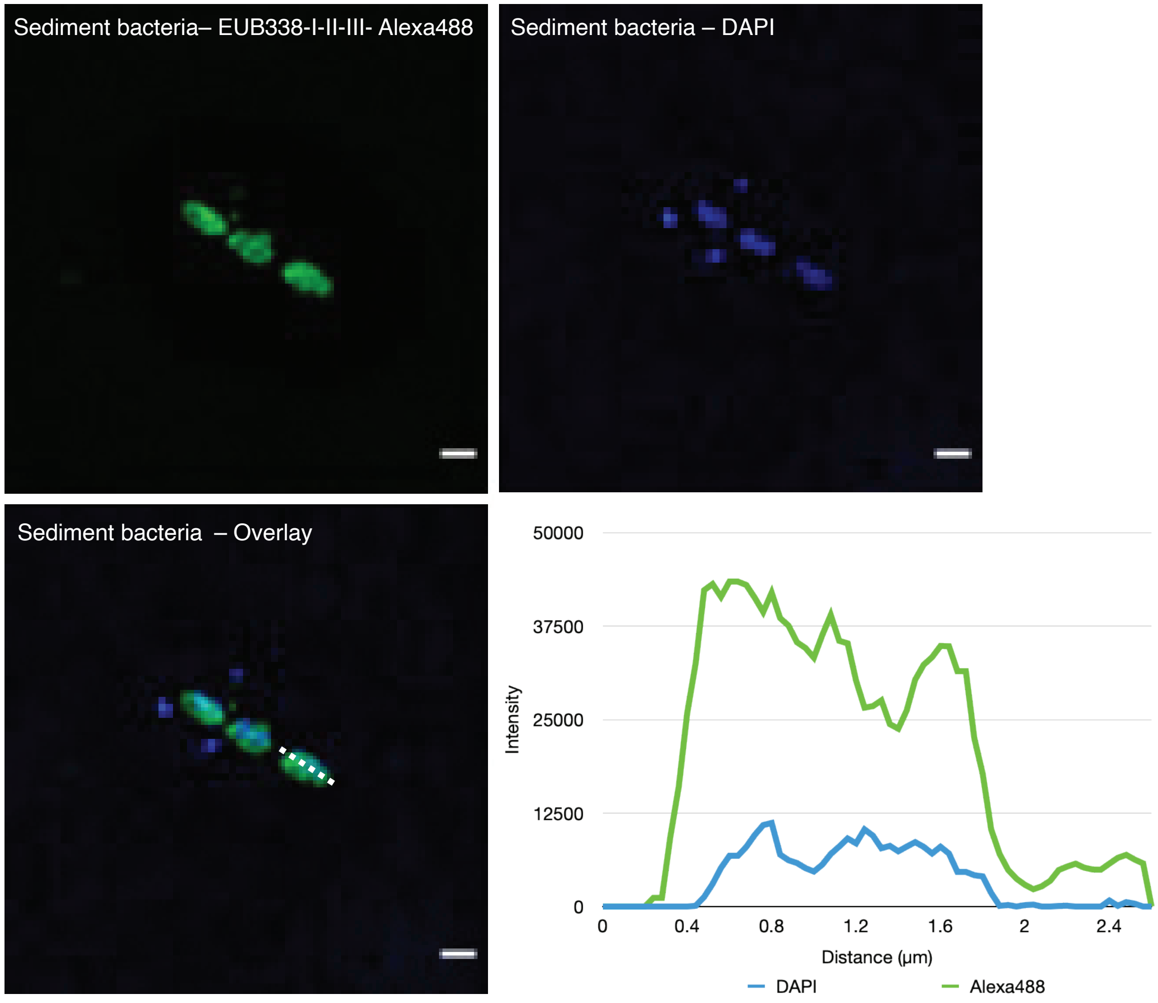
Sediment bacteria– EUB338-I-II-III- Alexa488
Sediment bacteria – DAPI
Sediment bacteria – Overlay
50000
37500
25000
12500
0
Intensity
0
0.4
0.8
1.2
1.6
2
2.4
Distance (μm)
DAPI
Alexa488
