# Supplementary material for: Spatial separation of ribosomes and DNA in Asgard archaeal cells
Source: ISME J. 2021 Aug 31;16(2):606–10. doi: 10.1038/s41396-021-01098-3 (PMC8776820; doi:10.1038/s41396-021-01098-3)
Supplement: Supplementary file 5 — Supplementary Fig. 4 [file 41396_2021_1098_MOESM5_ESM.pdf]

Supplementary Figure 4. Super-resolution structured illumination microscopy (SR-SIM) imaging of condensed DNA formation in *Escherichia coli* (A-B) and *Nitrosopumilus maritimus* cells (C-D). Single slices from the center of the focal plane are shown. Probe names and the dyes are indicated for each panel together with the intensity line profile of DAPI and FISH signals. Dashed lines indicate the position at which the fluorescence intensity profiles were recorded.

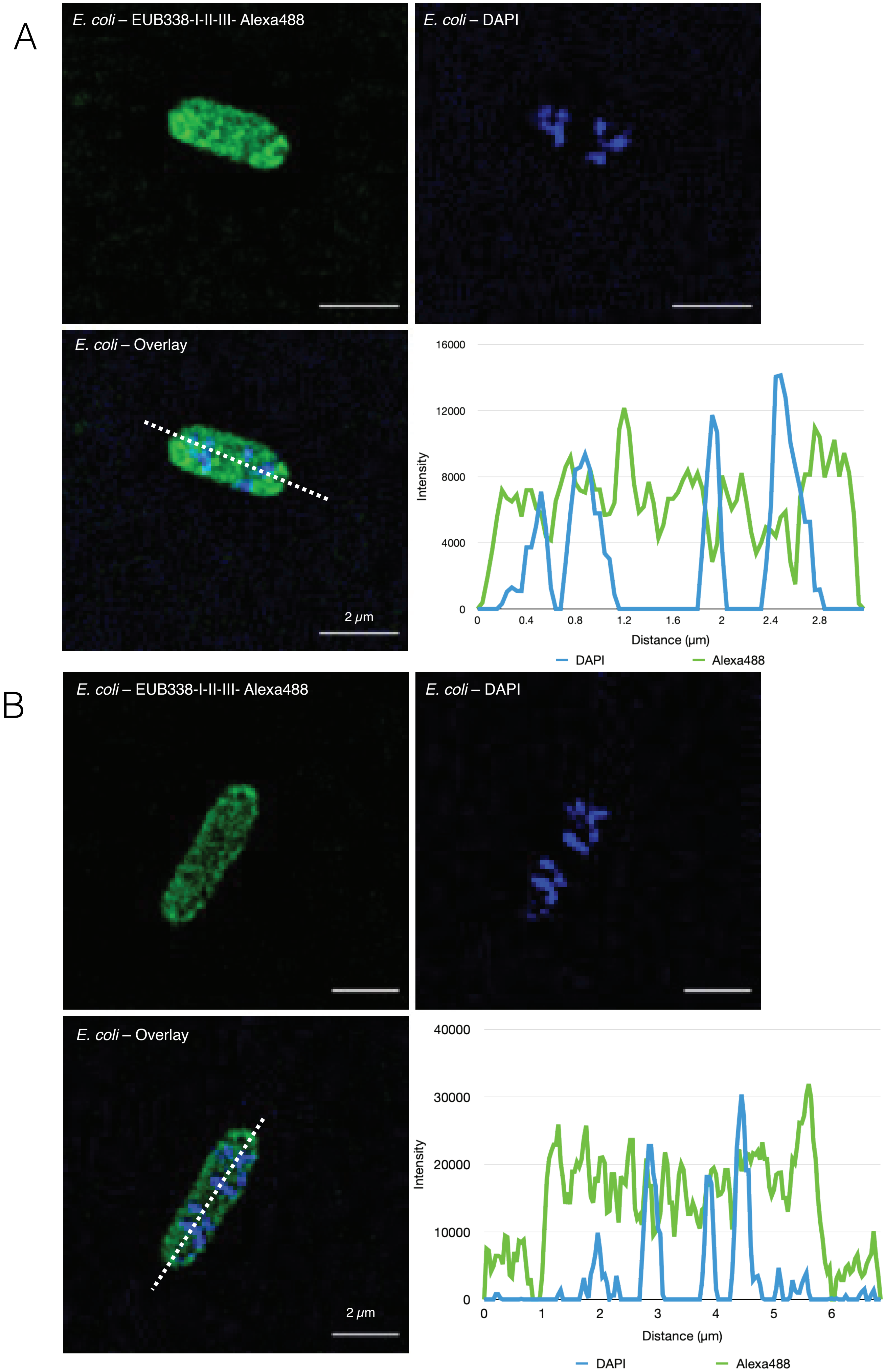
A
E. coli – EUB338-I-II-III- Alexa488
E. coli – DAPI
E. coli – Overlay
2 μm
16000
12000
8000
4000
0
Intensity
0
0.4
0.8
1.2
1.6
2
2.4
2.8
Distance (μm)
DAPI
Alexa488
B
E. coli – EUB338-I-II-III- Alexa488
E. coli – DAPI
E. coli – Overlay
2 μm
40000
30000
20000
10000
0
Intensity
0
1
2
3
4
5
6
Distance (μm)
DAPI
Alexa488

C

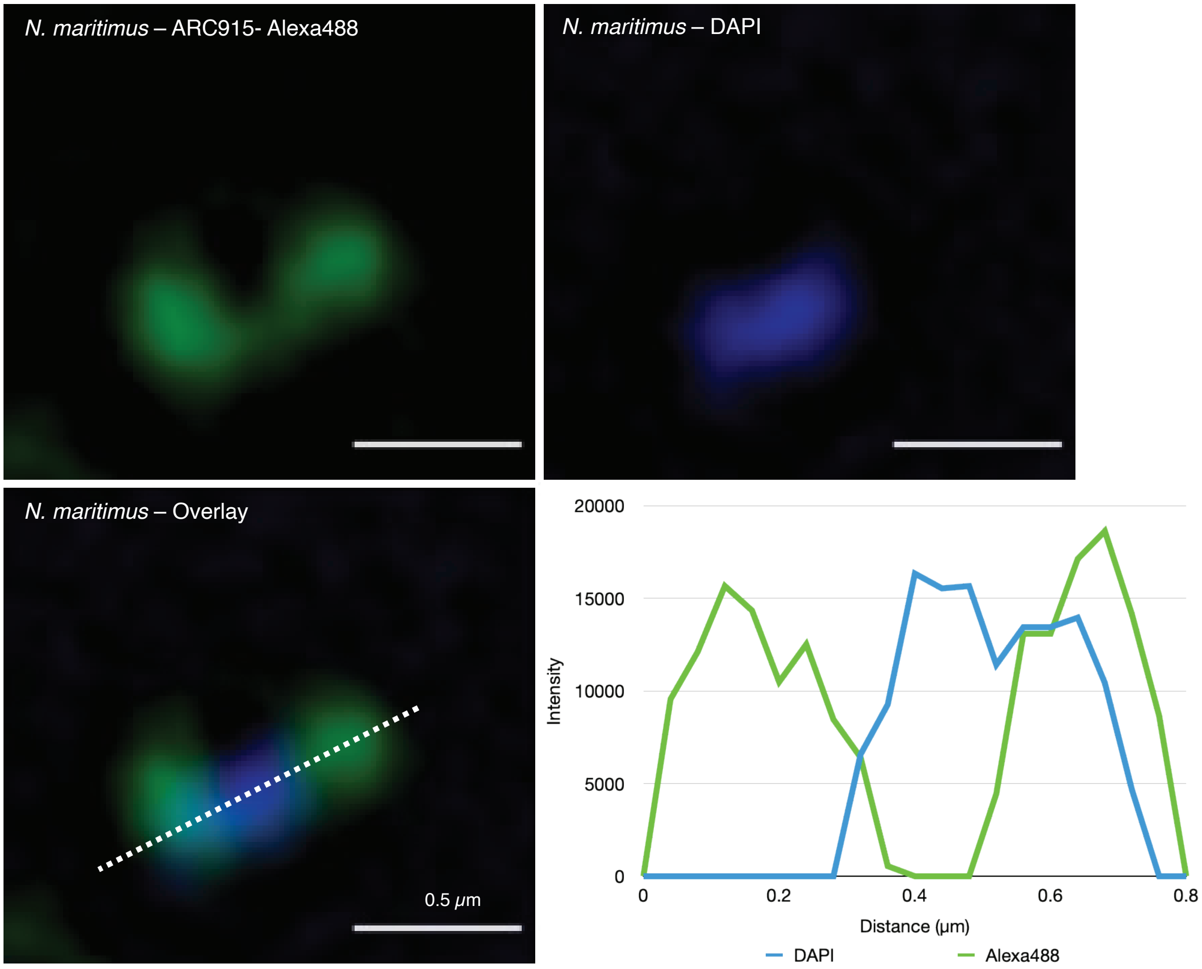
N. maritimus – ARC915- Alexa488
N. maritimus – DAPI
N. maritimus – Overlay
0.5 μm
20000
15000
10000
5000
0
Intensity
0
0.2
0.4
0.6
0.8
Distance (μm)
DAPI
Alexa488

D

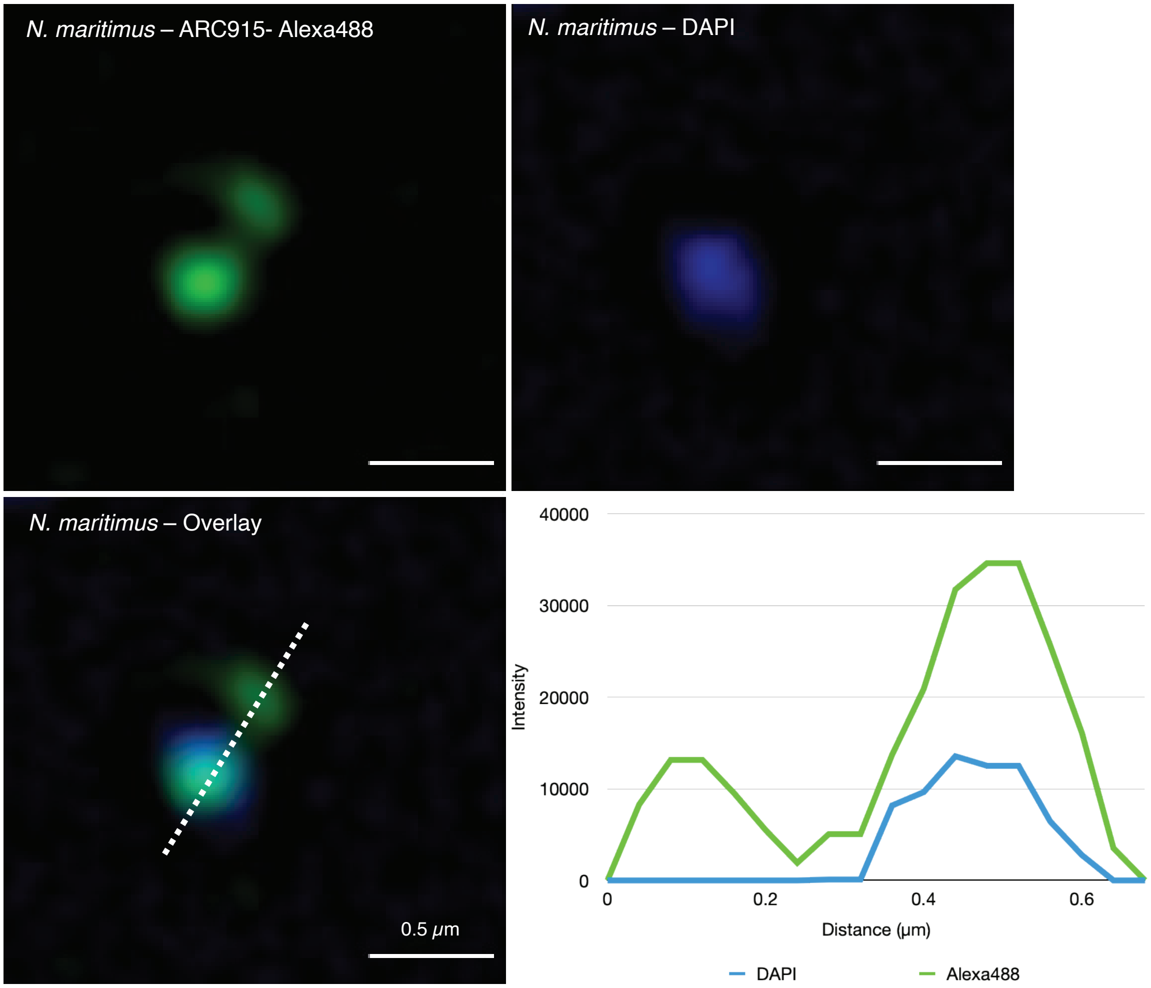
N. maritimus – ARC915- Alexa488
N. maritimus – DAPI
N. maritimus – Overlay
0.5 μm
40000
30000
20000
10000
0
Intensity
0
0.2
0.4
0.6
Distance (μm)
DAPI
Alexa488
