# Supplementary material for: Spatial separation of ribosomes and DNA in Asgard archaeal cells
Source: ISME J. 2021 Aug 31;16(2):606–10. doi: 10.1038/s41396-021-01098-3 (PMC8776820; doi:10.1038/s41396-021-01098-3)
Supplement: Supplementary file 6 — Supplementary Fig. 5 [file 41396_2021_1098_MOESM6_ESM.pdf]

Supplementary Fig. 5 Wheat germ agglutinin (WGA) staining of dual-labeled Loki- and Heimdallarchaeota cells. Probe or staining names and the dyes are indicated for each panel. Extracellular structures connected to the surfaces of Heimdallarchaeota cells are indicated with arrows. The axes that were used to measure cell sizes are demonstrated in dashed lines. Imaging was performed in super-resolution structured illumination microscopy (SR-SIM). Shown are single slice images taken from the center of the focal plane. The scale bar is 1 µm.

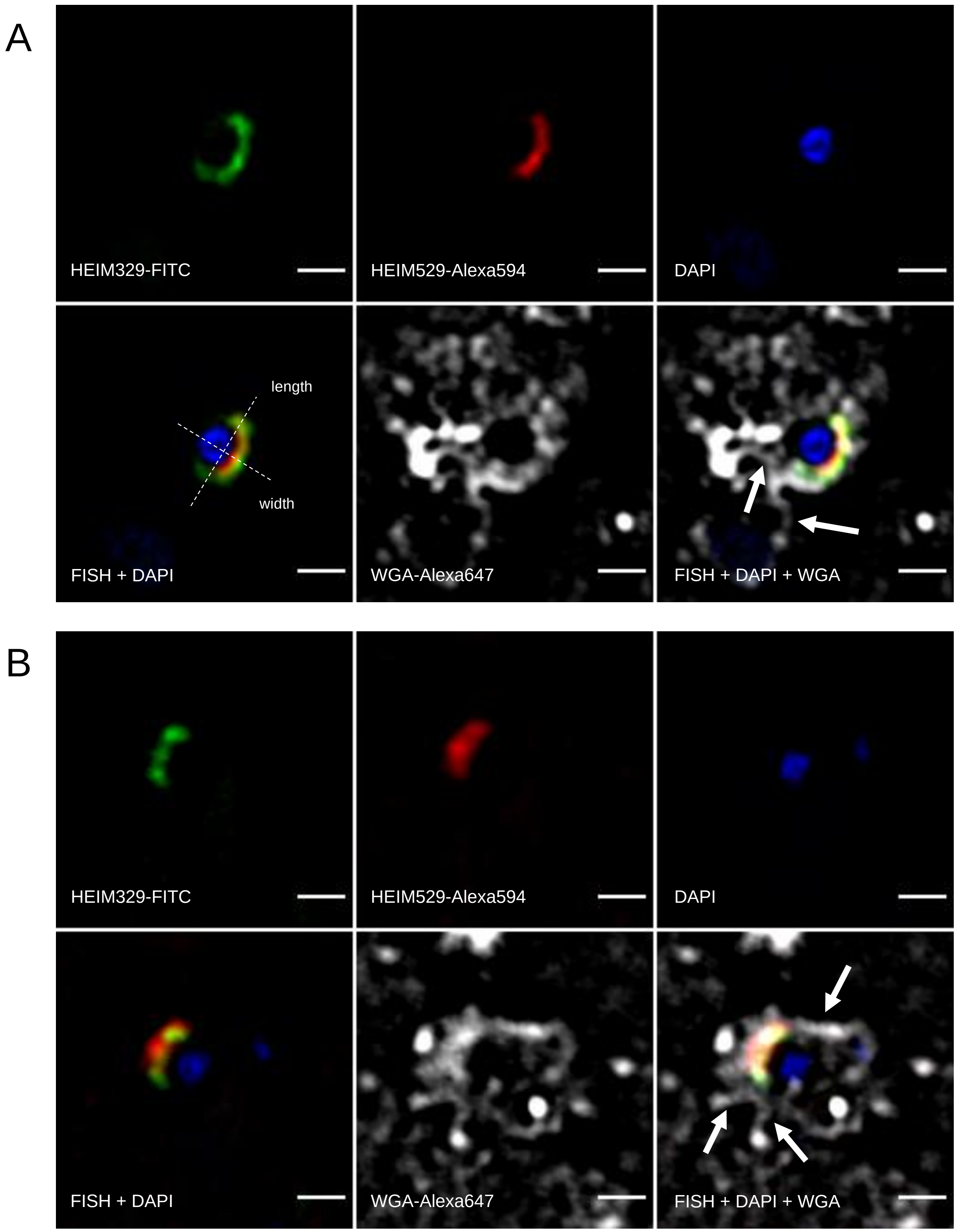
A
HEIM329-FITC
HEIM529-Alexa594
DAPI
length
width
FISH + DAPI
WGA-Alexa647
FISH + DAPI + WGA
B
HEIM329-FITC
HEIM529-Alexa594
DAPI
FISH + DAPI
WGA-Alexa647
FISH + DAPI + WGA

C

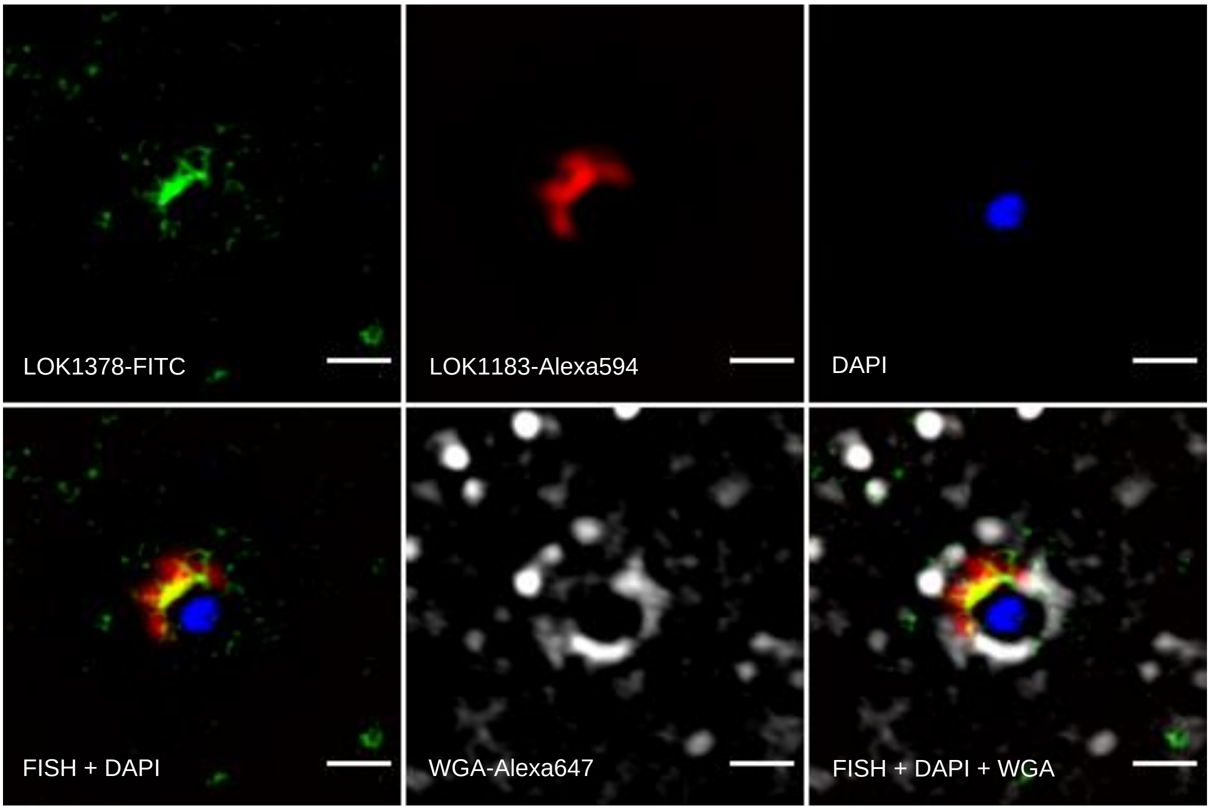
LOK1378-FITC
LOK1183-Alexa594
DAPI
FISH + DAPI
WGA-Alexa647
FISH + DAPI + WGA

D

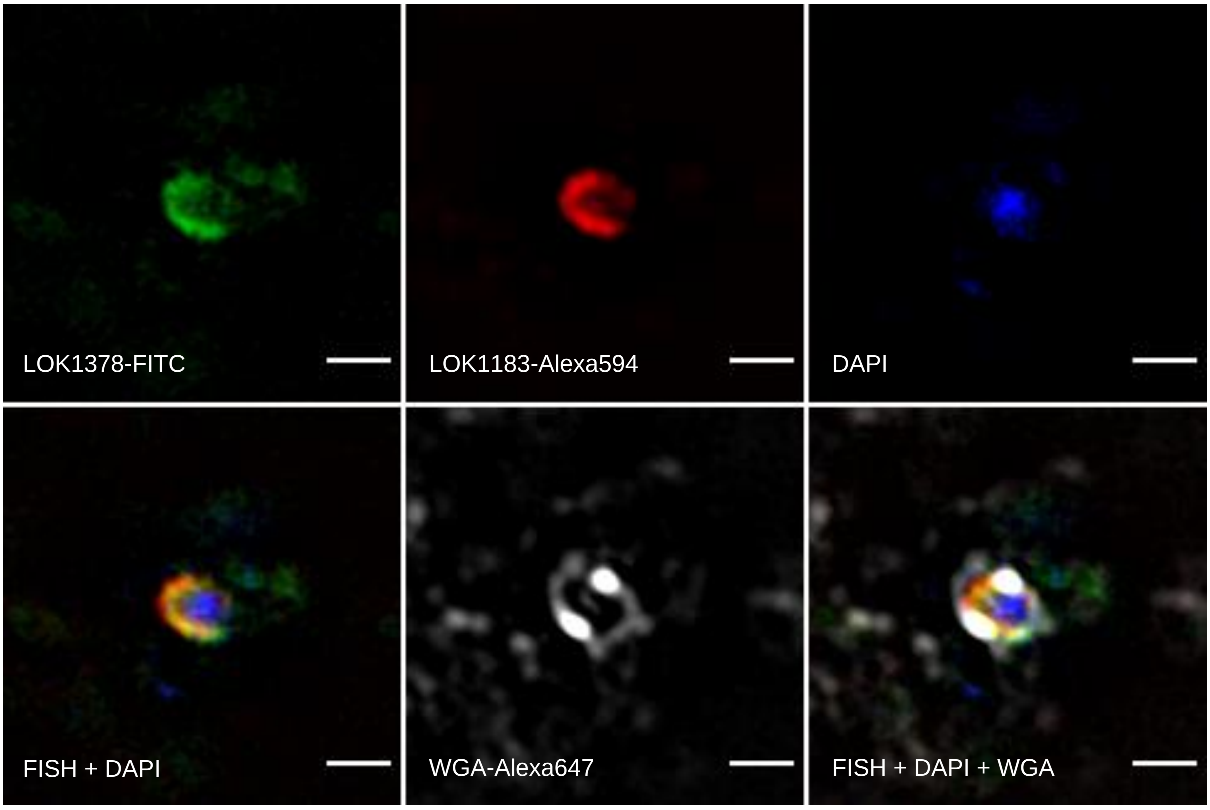
LOK1378-FITC
LOK1183-Alexa594
DAPI
FISH + DAPI
WGA-Alexa647
FISH + DAPI + WGA
