# Supplementary material for: Spatial separation of ribosomes and DNA in Asgard archaeal cells
Source: ISME J. 2021 Aug 31;16(2):606–10. doi: 10.1038/s41396-021-01098-3 (PMC8776820; doi:10.1038/s41396-021-01098-3)
Supplement: Supplementary file 7 — Supplementary Table 1 [file 41396_2021_1098_MOESM7_ESM.pdf]

A

Probe	Target	Probe sequence (5′ - 3′)	Formamide (%)	Reference
**LOK1183**	Lokiarchaeota	CTG ACC TGC CTT TGC CCG CT	20	This study
**LOK1378**	Lokiarchaeota	GTG TGC AAG GAG CAG AGA CG	20	This study
**HEIM329**	Heimdallarchaeota	GCA CTC GCA GAG CTG GTT TTA CC	20	This study
**HEIM529**	Heimdallarchaeota	CAC ACC GTA GTG CTA GGA CCA GT	20	This study
**ARC915**	Archaea	GTG CTC CCC CGC CAA TTC CT	35	Stahl and Amann, 1990
**EUB338 I**	Most Bacteria	GCT GCC TCC CGT AGG AGT	35	Daims et al., 1999
**EUB338 II**	Planctomycetales	GCA GCC ACC CGT AGG TGT	35	Daims et al., 1999
**EUB338 III**	Verrucomicrobiales	GCT GCC ACC CGT AGG TGT	35	Daims et al., 1999
**NON338**	Non-sense	ACT CCT ACG GGA GGC AGC	20	Amann et al., 1990

B

	SILVA		Aarhus Bay sediments (this study)		Non-target hits SILVA		
	Target group	Hits	Target group	Hits	0[a]	1[a]	2[a]
**LOK1183**	541	493	684	628	5	11	735
**LOK1378**	468	441	684	583	10	8270	10163
**HEIM329**	27	27	31	31	0	1	95
**HEIM529**	27	24	31	30	0	0	34

[a]**number of mismatches** between probe and non-target hit

C

Probe	Target	Probe sequence (5′ - 3′)	Formamide (%)
**c11LOK1183**	Competitor for LOK1183	CTG ACC TGC CGT TGC CCG CT	20
**LOK1183-h1**	Helper for LOK1183	CCT TCC TCM RTC TTA TCG A	20
**LOK1183-h2**	Helper for LOK1183	CAG GGG TTT CGG GGC ATA	20
**c16LOK1378**	Competitor for LOK1378	GTG TGC AAG GAG CAG GGA CG	20
**c20LOK1378**	Competitor for LOK1378	GTG TGC AAG GAG CAG GGA CA	20
**c2LOK1378**	Competitor for LOK1378	GAG TGC AAG GAG CAG GGA CG	20
**LOK1378-h1**	Helper for LOK1378	GTG GTG CTC CCC CGC CAA TT	20
**LOK1378-h2**	Helper for LOK1378	AGY ACA CTC GGA TGG AGC G	20
**HEIM329-h1**	Helper for HEIM329	GTC TKA GTG TCC TTC TCG GGG C	20
**HEIM329-h2**	Helper for HEIM329	CGC GCA TTG GGC ACA ATT CCT AA	20
**HEIM529-h4**	Helper for HEIM529	AGC CTT GCC CTC TGC TTG CTG TT	20
**HEIM529-h5**	Helper for HEIM529	CGG CTA CGG ATG CTT TAA GCC CA	20

Supplementary Table 1 Oligonucleotide probes used in this study. **A** Names, target groups, sequences, and formamide concentrations for newly designed Loki- and Heimdallarchaeota probes. **B** Number of the sequences in the target groups and the hits are shown together with non-target hits at 0,1 and 2 mismatches. For LOK1183 and LOK1378, only the sequences which contain the probe-target regions in SILVA database v132 (Quast et al. 2013) are considered. **C** Sequences and formamide concentrations for helper and competitor probes.
