# Supplementary material for: Spatial separation of ribosomes and DNA in Asgard archaeal cells
Source: ISME J. 2021 Aug 31;16(2):606–10. doi: 10.1038/s41396-021-01098-3 (PMC8776820; doi:10.1038/s41396-021-01098-3)
Supplement: Supplementary file 8 — Supplementary Table 2 [file 41396_2021_1098_MOESM8_ESM.pdf]

A

Cell ID	FISH	DAPI	Gap	Total width	Total length
Heimdall_18.8 5	0.6	0.7	0.12	1.25	1.52
Heimdall_18.8.2	0.58	0.72	0.11	1.37	1.61
Heimdall_11.19.3	0.65	0.55	0.173	1.26	1.27
Heimdall_11.19.5	0.7	0.8	0.2	1.79	1.24
Heimdall_25.11.1	0.64	0.67	0.23	1.42	1.45
Heimdall_13.12.11	0.56	0.55	0.19	1.27	1.07
Heimdall_10.12.2	0.45	0.34	0.24	1.1	1.28
Heimdall_10.12.3	0.6	0.42	0.16	1.11	1.7
Heimdall_10.12.5	0.35	0.58	0.05	1.04	1.5
Heimdall_10.12.6	0.47	0.62	0.28	1.4	1.05
Heimdall_13.12.8	-	-	-	1.32	1.4
**Average**	**0.56±0.1**	**0.60±0.14**	**0.18±0.07**	**1.30±0.20**	**1.37±0.21**

B

Cell ID	FISH	DAPI	Gap	Total width	Total length
Loki_89.5	0.49	0.39	0.26	1.07	1.24
Loki_89.3	0.55	0.52	0.07	1.09	1.43
Loki_89.2	0.8	0.5	0.07	1.23	1.43
Loki_89.7	0.63	0.6	0.54	1.8	1.6
Loki_89.6	0.96	0.47	0.15	1.5	1.67
Loki_89.4	-	-	-	1	1.53
Loki_11.19.5	-	-	-	1.4	1.56
Loki_11.19.4	0.91	0.5	0.09	1.49	1.73
Loki_12.11.2	0.6	0.56	0.18	1.23	1.68
Loki_12.11.2	0.61	0.58	0.2	1.34	1.59
Loki_25.11.1	0.48	0.47	0.17	1.07	1.14
Loki_25.11.3	0.78	0.53	0.07	1.3	1.58
Loki_15.12.3	0.46	0.42	0.05	0.91	1.5
Loki_15.12.1	0.5	0.46	0.16	1.25	1.16
Loki_15.12.5	0.44	0.47	0.12	1.11	1.64
Loki_25.11.5	-	-	-	1.05	1.32
Loki_25.11.4	-	-	-	1.28	0.74
Loki_15.12.4	-	-	-	1.7	1.25
**Average**	**0.63±0.18**	**0.49±0.06**	**0.16±0.13**	**1.27±0.24**	**1.43±0.25**

Supplementary Table 2 Measurement of **A** Heimdallarchaeota and **B** Lokiarchaeota cell sizes. Imaging was performed by super-resolution structured illumination microscopy (SR-SIM). Cell width was measured from the beginning of the FISH signal to the end of the DAPI signal, based on a line intensity profile taken along the narrow axis of the cell. Distance between DAPI and FISH staining was determined as the distance between FISH signal decay and the beginning of DAPI signal. The length was measured along the long axis of the FISH signal. The axes that were used for cell measurements were demonstrated in Supplementary Fig. 5. For the cells with central DAPI signals, only total width and total length are shown. All values are reported in µm.
